# Student motivation and instructional clarity: Linking experience sampling method data to objective behavioural observations

**DOI:** 10.1111/bjep.12775

**Published:** 2025-04-18

**Authors:** Alina Oschwald, Julia Moeller, Bärbel Kracke, Jaana Viljaranta, Julia Dietrich

**Affiliations:** ^1^ Institute of Educational Science Friedrich Schiller University Jena Jena Germany; ^2^ Institut für Bildungswissenschaften Leipzig University Leipzig Germany; ^3^ School of Educational Sciences and Psychology University of Eastern Finland Joensuu Finland; ^4^ Present address: Department of Psychology Philipps‐University of Marburg Marburg Germany; ^5^ Present address: Faculty of Philosophy and Education Catholic University of Eichstätt‐Ingolstadt Eichstätt Germany

**Keywords:** experience sampling method, instructional clarity, intensive longitudinal data, lecturer clarity, situated expectancy‐value theory

## Abstract

**Theoretical Background:**

Previous studies indicate that students' learning motivation varies across learning situations and is influenced by situational characteristics such as teaching behaviour. We focus on instructional clarity as one factor that may influence expectancies and task values.

**Aims and Research Questions:**

This study combines a previously published dataset of university students' experience sampling method (ESM) self‐reports about their current motivation with unpublished video recorded data of the same learning situations. We examined how lecturers' instructional clarity predicted states of students' learning motivation.

**Sample(s):**

One hundred and fifty‐five preservice teachers assessed their situated expectancies and task values three times within each weekly 90‐minute lecture over the period of 10 weeks. Simultaneously, video recordings of the lecturer were made and coded qualitatively for instructional clarity.

**Methods:**

We then combined students' motivation to lecturers' instructional clarity in the same learning situations. We used cross‐classified multilevel models to examine the associations of ESM surveys of students' motivation (level 1; *n* = 2227), nested in students (level 2a; *n* = 155), to ratings of lecturers' instructional clarity from videos (level 2b; *n* = 81).

**Results:**

Our findings indicated that none of the three indicators of instructional clarity (detail of explanation, variation of explanation and logical inconsistency) predicted global measures of motivation at the learning situation level. When exploring further into the facets of motivation, a detailed explanation predicted expectations of success and effort costs.

**Relevance:**

Overall, the idea of combining objective observation and subjective assessments emerged as valuable for adequately mapping complex dynamics in teaching–learning situations.

Recent studies show that university students' motivation varies between learning situations (e.g. Dietrich et al., 2017; Parrisius et al., 2022), raising the question of which characteristics shape situational learning motivation to ultimately help lecturers teach in the most motivating way. Motivation is influenced by lecturer‐related characteristics like enthusiasm (Frenzel et al., 2009; Keller et al., 2014) and formative assessment (Hondrich et al., 2018). This study delves into another key factor: the clarity of the instruction. Instructional clarity plays a central role, as it fosters students' persistence in learning (Pascarella et al., 2008), eases cognitive load, enabling more efficient information processing (Serki & Bolkan, 2024) and strengthens their academic motivation (Lazarides et al., 2019; Maulana et al., 2016). A Meta‐analysis found moderate‐to‐strong relationships between lecturers' clarity and students' learning (Titsworth et al., 2015). Yet its role in shaping students' situational learning motivation remains understudied.

## The situated expectancy‐value‐model

The situated expectancy‐value theory (SEVT) attempts to describe why and how students make achievement‐related decisions, show persistence and achieve performance in educational situations influenced by contextual, cultural and situated determinants (Eccles et al., 1983; Eccles & Wigfield, 2002, 2020). Accordingly, the core components of achievement motivation are task‐specific expectancies to succeed (e.g. ‘I will be good at these contents in the exam’) and task values (e.g. ‘I like these contents’).

Eccles et al. (1983); Eccles and Wigfield (2002) distinguish between *expectations of success*, the conviction of being able to solve a task in the future, and *competence beliefs*, a more stable and domain‐specific perception of one's academic self‐concept or abilities in a task. The task value, as outlined by Eccles et al. (1983); Eccles and Wigfield (2002), consists of four facets: *Intrinsic value* is characterized as the enjoyment of a task for its own sake. *Utility* pertains to the task's output for achieving short‐ or long‐term goals. *Attainment* describes the significance of success to identity and self‐concept. Perceived *cost* encompasses expected negatively valenced aspects of a learning situation, including three sub‐facets: negative emotions, missed opportunities and required effort.

Most empirical studies on SEVT have focused on inter‐individual differences. However, recent research has started to assess and model time‐ and context‐specific components of expectancies and values with an intra‐individual perspective and intensive longitudinal data capturing many learning situations (Dietrich et al., 2017, 2019; Malmberg et al., 2015; Martin et al., 2015; Moeller et al., 2020, 2022). Studies on situational and contextual determinants highlight variations in motivation across learning situations, topics and between days (e.g. Dietrich et al., 2017; Parrisius et al., 2022; Patall et al., 2016; Pöysä et al., 2018; Upadyaya et al., 2021). This includes changes in interests, competence beliefs, value beliefs and cost perceptions (Beymer et al., 2021; Parrisius et al., 2022; Tanaka & Murayama, 2014; Tsai et al., 2008).

## Instructional clarity

Eccles and Jacobs's (1986) socialization model expects both parents and teachers to shape student motivation through their behaviours and beliefs. This study focuses on lecturers' instructional clarity and its potential influence on situational measures of expectancies and values.

Teacher or lecturer clarity is a multidimensional construct (see Helmke, 2012; Lotz, Lipowsky, et al., 2013) that encompasses a lecturer's ability to explain content clearly (Stronge et al., 2011) by presenting content‐related aspects in a linguistically concise, coherent, comprehensible manner with technical accuracy (Lipowsky, 2020). This study focuses on instructional (explanatory) clarity (IC) as one dimension, which pertains to how lecturers deliver information. IC is reflected in their content statements (Lotz, Lipowsky, et al., 2013; Titsworth & Mazer, 2016). It differs from content coherence, which refers to the overall logical flow and conceptual consistency throughout the entire lesson (Lotz, Lipowsky, et al., 2013). IC involves well‐organized explanations, summarizing key points and using diverse methods such as examples and analogies to enhance student comprehension (Brophy, 2000; Cruickshank, 1985; Helmke et al., 2007; Lipowsky, 2020). Knowledge is most effectively developed when a range of examples is provided, giving students opportunities to apply concepts in various contexts (Titsworth & Mazer, 2016). In contrast, unclear lectures hinder schema development by omitting important details, presenting information in a confusing, incomplete, imprecise or circular manner and lacking context within a broader knowledge framework (Titsworth & Mazer, 2016; Wong et al., 2014). Based on this, we focus on three aspects of IC: (1) the amount of detail in the explanation (explanation in depth), (2) the amount of variation in the explanation (explanation in the width) and (3) the logical consistency of the argument.

IC helps students process information more effectively by presenting content in a structured and interconnected way, prioritizing depth over breadth (Brophy, 2000). It serves to build structures of knowledge (Brophy, 2000) and enhances persistence in learning (Pascarella et al., 2008; Rodger et al., 2007).

## How may instructional clarity affect cognitive and motivational processes?

Clear instruction reduces the difficulty and complexity of information, making it easier for learners to process and understand. This may boost learners' confidence in their ability to succeed and should foster a sense of competence, which in terms of Deci and Ryan's (1985) self‐determination theory is essential for maintaining motivation. IC strengthens students' perceived control beliefs (Fryer & Leenknecht, 2023) by giving them the sense that they better understand the material. A well‐structured lecture, guided by logically coherent instructions, may also direct learners' attention to key aspects and help streamline retrieval of information by focusing on relevant content during knowledge acquisition. The resulting support for working memory processes (Sweller et al., 1998) should be reflected in learners developing greater control beliefs over the learning material and higher expectations of success. Clear instructions should further reduce cognitive load, allowing students to focus on understanding the content rather than struggling with complex or unclear guidance. Research supports the idea that lower cognitive load enhances students' satisfaction and enjoyment (Hu et al., 2017) and thus helps maintain or increase intrinsic motivation (Serki & Bolkan, 2024). Students can engage more fully with the material, fostering a sense of satisfaction and enjoyment in the learning process. Moreover, when IC is high, learners can more easily process, store and retrieve information, facilitating the integration of new knowledge into existing mental schemas (Titsworth et al., 2015) and highlighting its practical applicability. This makes the learning process feel more meaningful and increase students' motivation to engage with the material. In conclusion, IC can be expected to foster students' competence beliefs and their expectations of success regarding their performance. Offering clear and structured guidance can additionally be expected to boost intrinsic, attainment and utility values.

Motivational costs arise when unclear or poorly structured instruction increases cognitive load (Feldon et al., 2019), making learning more difficult and frustrating. By simplifying complex material, IC should help learners to identify, discriminate and process the most important aspects, while minimizing unnecessary mental effort. It may prevent feelings of confusion, overwhelm or boredom, making the learning experience more satisfying and less taxing. In other words, higher IC can be expected to lead to lower perceived costs in students.

There are some research findings on the positive effects of teacher clarity on high‐school students' achievement, emotions and motivation (Chan et al., 2021; Lazarides et al., 2019; Maulana et al., 2016; Seidel et al., 2005; Simonton et al., 2017). While several studies indicate that teaching quality fluctuates (Decristan et al., 2017; Tsai et al., 2008; Voss et al., 2022), it remains largely unknown how situational changes in teacher or lecturer behaviour during a lesson relate to corresponding fluctuations in student motivation.

## Understanding within‐person change: Connecting subjective ESM self‐reports to more objective observations of a learning situation

From studies relying on time‐insensitive data and inter‐individual differences, intra‐individual dynamics cannot be derived (e.g. Dietrich et al., 2022; Moeller 2021; Molenaar, 2004; Reitzle & Dietrich 2019; Voelkle et al., 2014). Understanding the relationships among motivational states concurrently within a learning situation and their dependencies across learning situations requires time‐ and context‐sensitive assessments, analysed with intra‐individual methods.

In this study, student motivation and lecturer instructional behaviour were therefore assessed with intensive longitudinal data to capture short‐term changes within a lesson. To investigate momentary motivation, we resorted to the experience sampling method (ESM), which surveys self‐reports directly and immediately in real life (e.g. Hektner et al., 2007) and captures intra‐individual dynamics due to its high temporal resolution (Moeller et al., 2023). The dynamics of lecturer behaviour were assessed from moment to moment through observational video data, and IC was objectively coded as an environmental aspect of a specific learning situation. Merging self‐report ESM data with objective video data allows us to understand the external factors that may impact students' motivational experiences without shared method bias.

## Research question

The objective of this study was to examine how lecturers' instructional clarity predicted student motivation in a given learning situation. In the present data, student motivation varied on different levels. To examine our research question, our analysis focused on the motivational experiences within a learning situation that are shared by the students and differ from moment to moment. On this level of learning situations, we examined the assumption that the lecturer's IC in that moment would relate to the average motivation of the students at that same point in time. Specifically, we hypothesized that students would report higher expectancies (Hypothesis 1a), higher (intrinsic, attainment and utility) value (Hypothesis 1b) and lower perceived (emotional, opportunity and effort) cost (Hypothesis 1c) in those learning situations in which the lecturer presents content clearly (e.g. consistent in its logic and in a varied and detailed manner).

## METHODS

### Sample and procedure

This study is a secondary analysis of a previously published dataset (see Dietrich et al., 2017, 2019, 2022; Moeller et al., 2020, 2022; Reitzle & Dietrich 2019). Data were collected in the summer semester of 2014 at the University of Jena, Germany, in a lecture for student teachers. Student motivation was quantitatively assessed with an ESM design. Earlier publications focused on the variability in expectancies and values attributed to different learning situations, topics and individual student differences and the situational heterogeneity of motivation by investigating situational profiles. This study employed a mixed‐methods design, integrating qualitative coding of IC from video recordings of the lecturer. By combining the qualitative data with ESM data, this current analysis provides additional insights into the dynamic relationship between teaching behaviours and student motivation, thereby enhancing the understanding of the teaching‐learning context. The data set can be found in the online supplements.

**FIGURE 1:**
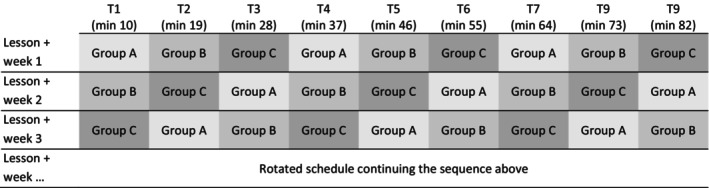
Schedule of ESM surveys rotated over three groups, so that each lesson got nine measurement time points, whereas each person was only surveyed three times per lesson. Shown is the design for the measurement of students' momentary motivation. There were three groups with alternating beeping schedules. Per lesson, there were nine ESM measurement time points, three per group and person (e.g. during a 90‐minute lecture, group A was interviewed at minute 10, 37 and 64; group B at minute 19, 46 and 73 and group C at minute 28, 55 and 82). The signalling schedule was rotated across the sessions. Illustration adapted from Moeller et al. (2020).

The study involved 155 first‐year university students (51% female, with an average age of 21.77 years, *SD* = 2.91, range: 19–46). Students reported their momentary motivation via smartphone (58–71% of the participants, *M =* 65% across 10 lessons) or paper‐and‐pencil questionnaire (29–42% of the participants, *M =* 35% across 10 lessons). Over the period of one semester, each student was surveyed three times per lecture over the course of 10 weekly lectures. To keep the burden on individual students low but maximise the number of available measurement time points per lecture, there were nine ESM surveys per lecture, distributed evenly across three groups with rotating survey schedules (see Figure 1). No group was beeped in the same signalling schedule in two subsequent lectures to avoid order effects and the effects of anticipations on the assessments (Bolger et al., 2003).

**FIGURE 2:**
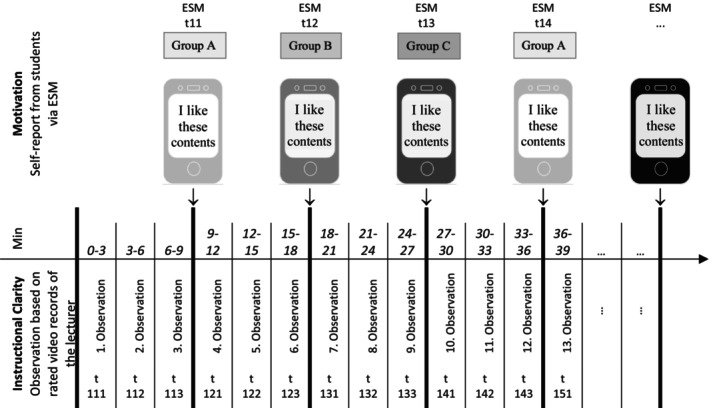
Study design for measuring instructional clarity. The coding intervals were 3 min long, so that three observations were submitted before every beep. This resulted in 27 observations per lecture. The first digit denoted the week, the second digit the timing of the beep in a lecture and the third digit the timing of the coding interval before a beep. For example, the abbreviation t123 represented the coding interval no. 3 (immediately before the beep) in the first week at the second beep. This observation was linked with the ESM survey of group B. While it marked the first beep for group B, within the lecture it was the second.

Parallel to the assessment by ESM surveys, video recordings of the lecturer (+ presentation slides) were made. These videotaped sessions were analysed with regard to IC by two independent raters using a qualitative coding manual. A time sampling schedule was used to rate the videos at three‐minute intervals, synchronized with ESM survey signals, that is, 3, 6 or 9 min before the signal (see Figure 2). A direct relationship between the code allocated in the interval and the responses to the signal could be established.

### Missing data

At the week‐level, only nine of the 10 sessions were considered because one session was conducted by a guest lecturer. Over the course of 9 weeks, a total of 27 time points were posed to each participant (three time points per session), with 81 time points distributed across the three groups (nine time points per session). Consequently, this resulted in 4185 possible measurement time points (155 participants × 3 ESM surveys × 9 weeks) at the ESM‐survey level.

Exclusions at the ESM‐survey level occurred for responses given more than 15 minutes after the beep, post‐lecture, if a person responded rapidly to the three surveys one after another or for uniform answers. By the end of the semester, participant numbers had decreased from 151 to 61 (*M =* 94.3 across the semester). In total, 2227 (53.21%) measurement points were considered as valid. To assess selectivity in the ESM data, we examined correlations between lecture attendance and variables such as gender, academic performance and student averages of situational motivation (see Dietrich et al., 2017). Higher attendance was related to academic performance (better grades; *r =* .33, *p <* .001) and lower average opportunity cost (*r =* .24, *p =* .010), but unrelated to gender (*r =* .04, *p =* .650).

The amount of observational data was affected by the occurrence of delayed or shortened lectures. Over the course of 9 weeks, a cumulative number of 243 observations were possible (27 per session), with 238 remaining for data analysis.

### Measures

#### Motivation

The students were requested to indicate their motivation during the previous 10 min by completing a questionnaire with 10 items, which had been adapted from various existing scales. This included eight items based on Eccles and Wigfield's (2002) SEVT and two items on situational effort and interests not considered in this study. See Dietrich et al. (2017) for validity details, Appendix A2 for item statistics and original wording in Appendix A1. *Situational expectancies* were assessed with two items asking about the expectation to be successful in the final exam (adapted from Wigfield & Eccles, 2000) and competence beliefs (Moeller et al., 2015). For example, students answered the question ‘I understand this content’. *Situational task values* were measured with six items from Gaspard et al. (2015), which were adapted to the context of ‘educational psychology’. Three items focusing on intrinsic value, attainment/personal importance and utility for future occupation. A sample task value item was: ‘It is important for me to know a lot about this content’. Cost components, including effort, emotional and opportunity costs, were measured separately. Students responded on a four‐point Likert scale ranging from 1 = *does not apply* to 4 = *fully applies*. To represent average perceptions, composite scores for situational expectancies, situational values and situational costs were created at the ESM‐survey level based on item means.

#### Instructional clarity

The video recordings of the lecturer were analysed using a high‐resolution time sampling design with three‐minute observation units to capture the dynamics of IC. The start of the coding was synchronized with the time stamps of the video, which corresponded to the beeps of the motivational data.

##### Development of the coding manual

A high‐inference rating system (Lotz, Gabriel et al., 2013) was developed, blending deductive and inductive approaches (Hugener et al., 2006; Lotz, Lipowsky, et al., 2013; Seidel et al., 2003). A theoretically and empirically defined notion of IC, along with distinct classification rules, formed the starting point. The coding manual was refined using video material that was not included in further analyses until moderate inter‐rater reliability was achieved (based on the interpretation of Koo & Li, 2016). All indicators reached the requirements for sufficient data quality (95% confidence interval: detail (ICC = .62–.75), variation (ICC = .55–.70) and logical inconsistency (ICC = .53–.69)). For details on inter‐rater reliability, see Appendix B4.

Our investigation focused on aspects of content clarity, which encompasses how content is conveyed in discrete lesson segments through the utterances and actions of the lecturer. Utterances that contain logically coherent and comprehensible representations, highlight and summarize key points and provide varied explanations (e.g. using illustrations, examples and analogies) serve an important function in fostering students' comprehension (Brophy, 2000; Cruickshank, 1985; Helmke et al., 2007; Lipowsky, 2020). Students can process information more easily when lecturers present the internal structures of content in a meaningful manner, depicting it as a network of interrelated information and prioritize on in‐depth coverage over breadth (Brophy, 2000).

##### Coding manual for instructional clarity

The rating system comprised three items: two positive indicators measuring the amount of detail (depth) and variation (width) in the explanation, and one negative indicator measuring the logical consistency of the argumentation (hereinafter referred to as logical inconsistency). All indicators were applicable to both abstract content and examples, always focusing on the central content rather than secondary topics mentioned. If the positive indicators were fulfilled and the negative indicator was not fulfilled, this represented an ideal performance in terms of IC. Initially, an overall rating was planned based on the three indicators, but we abandoned this idea due to the low variance of the negative indicator (see Table 1). For more details about the rating system, the coding process, the item statistics and the inter‐rater reliability, refer to the Coding Manual in Appendix B.

**TABLE 1:** Descriptive item statistics across all intervals.

Item	*n*	*M*	*SD*	*MSSD*	*r*	Absolute and relative frequencies		
						0 = not/never observed	1 = partly observed	2 = continuously observed
Detail	238	1.09	.68	.74	.19	46 (18.93%)	125 (51.44%)	67 (27.57%)
Variation	238	.73	.62	.56	.26	87 (35.80%)	129 (53.09%)	22 (9.05%)
Logical inconsistency	238	.18	.40	.26	.17	196 (80.66%)	41 (16.87%)	1 (.41%)

Abbreviations: M, mean; MSSD, mean square successive difference; *n*, video observations; *r*, autocorrelation; SD, standard deviation.

### Analytic strategy

**FIGURE 3:**
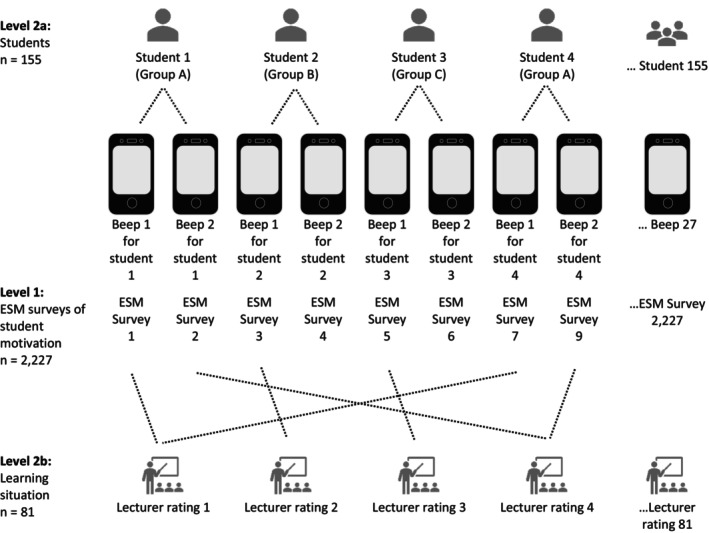
Structure of the two‐level cross‐classified data. Each ESM survey is hierarchically nested within both a specific student and a particular time point of the learning situation, demonstrating the cross‐classified structure of the dataset. For example, ESM Survey 1 represents the momentary motivation of Olivia (Student 1) during the first learning situation (Beep 1 for Olivia). Similarly, ESM Survey 7 captures John's (Student 4) momentary motivation at the same learning situation (also Beep 1 for John). Both ESM surveys account for the same learning situation (Lecturer Rating 1), reflecting the momentary motivation in response to the specific behaviour observed from the lecturer at that time point.

Each motivational state at a given moment resulted from a unique combination of the personal characteristics and the teaching situation. To fit the structure of the data accurately, we applied cross‐classified multilevel regressions (CCMM). This modelling strategy was considered appropriate for imperfect hierarchies (Snijders & Bosker, 2012) and for longitudinal data because repeated measurements in continuous samples can be nested within individuals (Hox et al., 2018; Nezlek, 2007). A lower‐level unit (ESM survey) was nested in two higher‐level units of different types (here students and learning situation, see Figure 3); nevertheless, the higher‐levels are not nested in either direction (Snijders & Bosker, 2012).

The motivational response of each individual person at a given time point $Y_{i\left(t,j\right)}$ was nested within the cross‐classification of a student *j* and learning situation *t* (see Figure 3). In this sense, *level 2a* is characterized by every individual student *j* (index *j* runs from 1 to J/155).

The *level 2b* represents the objective characteristics of the learning situation that comprise the teaching behaviour *t* at the time of measurement. In this way, the index *t* represents the temporal course of the lecture and runs from 1 to T/81.

*Level 1* reflects the individual ESM surveys and describes the motivational response *i* of a person *j* at a certain learning situation *t* and runs from 1 to I/2227. We fitted the following cross‐classified multilevel model (CCMM) with a single covariate by using the notation of Hox et al. (2018):

(1) $$Y_{i\left(t,\;j\right)}=\gamma_{00}+\gamma_{10}\cdot \text{instructional clarity}\;t+u_{0t}+v_{0j}+e_{i\left(\mathrm{tj}\right)}$$

(2) $$\begin{array}{c} Y_{i\left(t,\;j\right)}=\gamma_{00}+\gamma_{10}∙\text{instructional clarity}:\text{detail}\;t+\gamma_{20}∙\text{instructional clarity}:\text{variation}\;t+\\ \gamma_{30}∙i\text{nstructional clarity}:\text{logical consistency}\;t+u_{0t}+v_{0j}+e_{i\left(\mathrm{tj}\right)} \end{array}$$

According to this equation, the total variance of the motivational state can be decomposed into one within‐level and two between‐levels. This procedure permitted the examination of person‐situation relations in learning situations by disentangling situational, personal and contextual influences (Nezlek, 2007; Moeller et al., 2020).

The fixed intercept $\gamma_{00}$ refers to the mean motivation of all students across all learning situations. The random part of the intercept $u_{0t}$ relates to the random effect for time points and corresponds to the inter‐individual average motivation of all students in a certain situation. The random part of the parameter $v_{0j}$ refers to the random effect for persons in terms of a stable person‐specific mean value of motivation over time. $e_{i\left(\mathrm{tj}\right)}$ denotes the measurement‐specific residual for the motivational state which corresponds to the person‐ and situation‐specific deviation from the group mean. Equation 1 contains a covariate $\gamma_{10}$ on the learning situation‐level 2b, in which the slope of the parameter equals the average effect of IC. In Equation 2, each predictor – Detail ($\gamma_{10}$), Variation ($\gamma_{20}$) and Logical Consistency ($\gamma_{30}$) – represents the effect of a specific aspect of IC on students' inter‐individual mean motivation at a given moment. IC is characterized by varying over time, but not between students and thus reflecting time‐varying characteristics of the context (Hosoya et al., 2014). Since the video observations had a higher resolution, we averaged the three observations intervals prior to a beep. Due to the lack of variance in logical inconsistency, we recoded the variable to 0 = *did not occur*, 1 = *occurred* before calculating the average score.

The present study aimed at determining the proportion of motivational variance that was localized at the learning situation level. The student level essentially fulfilled the function of controlling the effects on the other two levels.

Bayesian estimation techniques were chosen for the analysis. The data were prepared in R (R Development Core Team, 2008); statistical analyses were performed using M*plus* version 8.8 software program (Muthén & Muthén, 1998‐2017). An overview of all calculated models and prior settings can be found in the model documentation (Appendix C).

## RESULTS

### Descriptive statistics

#### Situational motivation

The main analyses referred to the composite SEVT components: situational expectancies, situational (positive) values and situational costs rather than the single facets of the SEVT components. Table 2 (and see Appendices A.3 and A.4 for details) presents the results of an unconditional CCMM (M0) that decomposed variance of students' motivation into three variance components: one within‐level (ESM‐survey‐level: *n* = 2227) and two between‐levels (student‐level: *n* = 155; learning situation‐level: *n* = 81). The bulk of variability in *situational motivation* was on the intra‐individual, situation‐specific ESM survey level 1, which accounts for 39.7%–62.7% of the total variance. The between‐level variance in motivation was driven largely by students (33.6%–57.1%) and less by learning situations (3.2%–4.3%). As expected, expectancies and values were positively associated (L1: *r* = .51, L2a: *r* = .58, L2b: *r* = .86), while costs were negatively associated with expectancies (L1: *r* = −.27, L2a: *r* = −.46, L2b: *r* = −.88) and values (L1: *r* = −.30, L2a: *r* = −.30, L2b: *r* = −.87). This correlation structure was found at all levels and was highest on learning situation‐level. As additional information we provide the descriptive statistics for students' motivation separated for lectures in Appendix A.2.

**TABLE 2:** Unconditional cross‐classified multilevel model (M0).

	Estimates [*CI*]
Means	
Expectancies	3.099
Task values	2.991
Costs	1.792
Variances	
L1 expectancies	.166 [.155; .177]
L1 task values	.177 [.166; .189]
L1 costs	.166 [.156; .178]
L2a expectancies	.089 [.066; .120]
L2a task values	.130 [.100; .172]
L2a costs	.240 [.186; .312]
L2b expectancies	.010 [.005; .017]
L2b task values	.014 [.008; .023]
L2b costs	.013 [.008; .022]
ICC	
L2a expectancies	.336 [.272; .407]
L2a task values	.405 [.340; .476]
L2a costs	.571 [.506; .638]
L2b expectancies	.037 [.020; .062]
L2b task values	.043 [.025; .070]
L2b costs	.032 [.019; .051]

Abbreviation: CI, credibility interval.

#### Instructional clarity

Appendix B.2 presents the means and the standard deviations of the video observations. During the lectures, the lecturer explained the content mainly in *detail*, with less *variation*. In all sessions, the lecturer's argumentation was *consistent* in its *logic*. Overall, these results suggest that IC was high in the majority of situations, but the average differed from lecture to lecture. Furthermore, we calculated inter‐item correlations at the level of learning situations (i.e. mean of three observation intervals prior to a beep) because this equals the level on which covariates were included in the conditional CCMMs. Detail of explanation and logical inconsistency were negatively associated (*r* = −.27, *p =* .016), whereas the variation of explanation was uncorrelated with detail of explanation (*r* = .04, *p =* .751) and logical inconsistency (*r* = −.19, *p =* .095).

### What is the link between fluctuations in lecturers' instructional clarity and students' situational motivation?

Table 3 presents the results of three CCMMs that included the indicators of IC as predictors to explain the variance at the learning situation‐level (for zero‐order correlations see Appendix C.1, Table C.1.1). When adding the learning situation‐level covariates to the models, the variance within the situation‐specific, intra‐individual ESM‐survey‐level and the between learning situation‐level variance were nearly identical compared to the null model (Table 2). Contrary to our hypotheses, all three indicators of IC (detail of explanation, variation of explanation and logical inconsistency) neither predicted expectancies nor task values or costs at the learning situation‐level.

**TABLE 3:** Cross‐classified multilevel analysis results for predicting students' situational motivation (SEVT components) by instructional clarity.

	Model M1	Model M2	Model M3
	Expectancies	Values	Costs
	Estimates [CI]	Estimates [CI]	Estimates [CI]
Fixed effects			
Intercept	3.097 [2.993; 3.202]	2.977 [2.855; 3.100]	1.785 [1.657; 1.917]
Learning situation‐level			
Detail	.032 [−.030; .093]	.012 [−.060; .083]	−.026 [−.095; .043]
Variation	−.015 [−.079; .051]	.015 [−.060; .090]	.045 [−.027; .118]
Logical Inconsistency	−.065 [−.183; .052]	−.037 [−.172; .097]	.005 [−.127; .134]
Random parameters			
$\sigma^{2}$ ESM‐survey‐level	.166 [.155; .177]	.177 [.166; .189]	.166 [.156; .178]
$\sigma^{2}$ student‐level	.084 [.063; .113]	.125 [.096; .165]	.233 [.183; .303]
$\sigma^{2}$ learning situation‐level	.009 [.004; .015]	.013 [.008; .022]	.012 [.007; .020]
ICC			
Student‐level	.324 [.263; .394]	.397 [.332; .467]	.566 [.503; .631]
Learning situation‐level	.033 [.017; .058]	.042 [.024; .069]	.030 [.017; .049]
*ppp*‐value	.498 [−12.381; 13.129]	.497 [−12.313; 13.116]	.496 [−12.267; 13.507]

*Note*: This Table presents the results for three cross‐classified multilevel models for each SEVT component: expectancies, values and costs and includes IC as learning situation‐level covariates. IC was averaged across three observation intervals prior to a beep. For the fixed effect estimates, cell entries are parameter (beta) estimates and CI = 95% credibility intervals. Random effects are presented as estimates and credibility intervals. The *ppp*‐value refers to posterior predictive *p*‐value, a measure of model fit.

In a further step, we examined CCMMs (see Table 4) with individual SEVT facets (e.g. expectation of success and competence beliefs). The models with individual SEVT facets explained a notable proportion of variance at the within ESM‐survey level (up to .229 explained variance in expectations, .304 in values and .366 in costs) and at the between‐student level (up to .103 explained variance in expectations, .183 in values and .406 in costs). The explained variance at between‐learning situations was low (up to .016 explained variance in expectations, .022 in values and .024 in costs). As Table 4 shows, no significant associations with the IC indicators were identified in any of the models.

**TABLE 4:** Cross‐classified multilevel analysis results for predicting students' situational motivation (SEVT facets) by instructional clarity.

	M1.a expectation of success	M1.b competence belief	
	Estimates [*CI*]	Estimates [*CI*]	
Fixed effects			
Intercept	2.901 [2.799; 3.005]	3.295 [3.163; 3.425]	
Learning situation‐level			
Detail	.080 [.020; .140]	−.014 [−.093; .064]	
Variation	.005 [−.058; .068]	−.034 [−.117; .050]	
Logical Inconsistency	−.009 [−.124; .106]	−.113 [−.261; .037]	
Random parameters			
$\sigma^{2}$ (ESM‐survey‐level)	.220 [.206; .235]	.229 [.215; .245]	
$\sigma^{2}$ (student‐level)	.099 [.074; .133]	.103 [.077; .140]	
$\sigma^{2}$ (learning situation‐level)	.006 [.002; .012]	.016 [.009; .026]	
ICC			
Student‐level	.306 [.245; .374]	.297 [.236; .365]	
Learning situation‐level	.017 [.005; .037]	.045 [.026; .074]	
ppp‐value	.482 [−11.722; 11.972]	.496 [−12.049; 12.953]	

	M2.a Intrinsic value	M2.b Attainment value	M2.c Utility value
	Estimates [CI]	Estimates [CI]	Estimates [CI]
Fixed effects			
Intercept	2.944 [2.793; 3.095]	2.881 [2.755; 3.011]	3.106 [2.971; 3.240]
Learning situation‐level			
Detail	.024 [−.070; .116]	.018 [−.055; .089]	−.003 [−.082; .075]
Variation	−.030 [−.128; .067]	.029 [−.048; .105]	.051 [−.033; .133]
Logical Inconsistency	−.016 [−.190; .157]	−.035 [−.173; .103]	−.055 [−.204; .094]
Random parameters			
$\sigma^{2}$ (ESM‐survey‐level)	.304 [.285; .325]	.273 [.256; .292]	.268 [.251; .286]
$\sigma^{2}$ (student‐level)	.114 [.084; .155]	.183 [.141; .241]	.155 [.118; .207]
$\sigma^{2}$ (learning situation‐level)	.022 [.013; .037]	.010 [.004; .019]	.014 [.007; .025]
ICC			
Student‐level	.259 [.203; .323]	.393 [.328; .464]	.355 [.292; .425]
Learning situation‐level	.050 [.029; .083]	.021 [.008; .041]	.032 [.017; .056]
ppp‐value	.483 [−11.457; 11.987]	.492 [−11.417; 12.284]	.487 [−12.673; 12.787]

	M3.a Effort cost	M3.b Emotional cost	M3.c Opportunity cost
	Estimates [*CI*]	Estimates [*CI*]	Estimates [*CI*]
Fixed effects			
Intercept	1.955 [1.783; 2.129]	1.618 [1.480; 1.759]	1.770 [1.624; 1.923]
Learning situation‐level			
Detail	−.068 [−.167; .030]	.002 [−.074; .080]	−.023 [−.094; .050]
Variation	.091 [−.012; .196]	.013 [−.070; .095]	.021 [−.054; .097]
Logical Inconsistency	.034 [−.154; .219]	−.071 [−.224; .078]	.058 [−.079; .193]
Random parameters			
$\sigma^{2}$ (ESM‐survey‐level)	.366 [.343; .391]	.283 [.265; .302]	.266 [.249; .284]
$\sigma^{2}$ (student‐level)	.304 [.235; .398]	.197 [.151; .261]	.406 [.319; .527]
$\sigma^{2}$ (learning situation‐level)	.024 [.014; .040]	.013 [.006; .024]	.010 [.004; .019]
ICC			
Student‐level	.438 [.373; .507]	.400 [.335; .470]	.595 [.533; .658]
Learning situation‐level	.035 [.020; .057]	.026 [.013; .048]	.014 [.006; .028]
ppp‐value	.490 [−12.135; 13.129]	.485 [−12.067; 12.537]	.489 [−13.164; 12.242]

Building on this, we further split the data into individual observation intervals (first, second and third interval prior to a beep) and examined supplementary CCMMs for each SEVT facet (e.g. expectation of success and competence beliefs; results in Appendices C.2–C.4). We found that only the clarity facet detail of explanation predicted lower perceived costs (*β =* −.047), particularly the facet effort cost (*β =* −.087), and expectation of success (*β =* .068) in the first observation interval, and continued to predict variability in expectation of success (*β =* .041) in the third observation interval. This indicates that situational expectations of success were higher, while situational costs were lower, when the lecturer presented the content in detail. Variation in explanation and logical inconsistency were, contrary to our hypotheses, not related to any of the facets of students' situational motivation.

## DISCUSSION

A growing body of research has highlighted the need to focus on the situational complexity of motivation (Eccles & Wigfield, 2020; Dietrich et al., 2017; Nolen, 2020). To advance our understanding of students' motivation dynamics, we investigated its association with a lecturer's instructional clarity. More precisely, our research focus was on the motivational experiences within a learning situation that are shared by all students attending the lecture, reflecting the at least partial correspondence among the subjective motivations of several students.

Our findings suggested that only a small part of the changes in motivation could be ascribed to differences in the (shared) learning environment from moment to moment. We then questioned whether instructional clarity explained this variation in the average motivation of all students at a given moment. Contrary to our hypotheses (1a, 1b, 1c), all three indicators of IC, that is, detail of explanation, variation of explanation and logical inconsistency, did not predict expectancies, nor task values and costs at the learning situation‐level.

This indicates that IC as a situational characteristic, in the sense of an objective component, had no impact on the shared experience of expectancies, values and costs in a given situation. Our situation‐specific findings thus contradict previous findings, where perceived teacher clarity was crucial for motivational changes and achievement (Lazarides et al., 2019; Maulana et al., 2016; Rodger et al., 2007), and for high school student control and value beliefs (Simonton et al., 2017). However, the evidence seems to be quite diverse, as the effects on student learning were extremely heterogeneous across the studies examined in middle school, high school and college (Titsworth et al., 2015), due in no small part to the imprecise definition and multidimensionality of lecturer clarity.

One explanation for our results is that students share only little variance in motivation at the learning situation level, suggesting that they did not share a common perception of IC. Another reason could be a lack of true variance in IC. Averaging the three observations before the beep equalizes the erstwhile high and low expressions of IC, resulting in the mean observations being more similar. Therefore, we performed the additional analyses with a higher temporal resolution. Non‐linear associations might also explain our findings. In particular, too little and too much detail could be experienced as demotivating. An overabundance of IC, for example excessive repetition, can be frustrating or annoying (Titsworth et al., 2015). Furthermore, task values could be high (e.g. due to exam‐relevance) even if they are not completely comprehensible. Some initial evidence pointed out that forming expectations is complex (Reinhard & Dickhäuser, 2009) and that performance expectancies predict performance only in challenging tasks (Marshall & Brown, 2004). IC may only be relevant in expectancy formation when a task is perceived as difficult.

Even though our main hypotheses could not be confirmed, upon closer examination, we found that specifically detail of explanation mattered for students' expectancy of success, effort cost and costs overall. These associations, however, were mainly observed at specific time intervals: particularly detailed explanation up to 9 min before the beep seemed to be relevant for motivation. While this, on the one hand, points to the need for replication to rule out that any effects are unstable, it is possible that the finding is suggestive of a substantive interpretation. It might be that some kind of ‘sleeper’ effect operates such that students must cognitively process a detailed explanation before it can impact their motivational appraisals. Therefore, we might interpret the exploratory findings of this study as initial evidence for temporal delays in cognitive processing that precede motivational appraisals.

### Shared perception vs. individual perception of instructional clarity

As we observed that students did not share much variance in motivation at the learning situation level, it is possible that they didn't share either a common perception of IC in this context. Our results do not rule out that IC in general has no effect on motivation. In our study, IC does not relate to motivation in the same way for, let's say, John as for Olivia. But at the person‐ and situation‐specific within‐level (ESM‐survey level), John might perceive the lessons as clear, while Olivia does not. Students of the same lecture perceive the teaching of the same lecturer in a very heterogeneous way (Bardach et al., 2021).

Although there might be no overall students' perception of IC, there could be a subjective students' perception, which depends on stable or fluctuating student characteristics such as prior knowledge (Moeller et al., 2020). This idea aligns with the results of the variance decomposition and earlier findings (Parrisius et al., 2022). Motivation is directly influenced by the situation (indicating situative nature), but this influence varies among students (indicating subjectivity) due to higher proportions of variance localized at the ESM survey level as compared to between learning situation level. Situational perception varies between individuals and can even differ within the same situation (Horstmann et al., 2021; Ziegler et al., 2019). These differences in situation perception are associated with momentary fluctuations in achievement motivation states (Witte et al., 2024). Individual perceptions are thus likely to have a stronger impact on motivation than the objective clarity of the instruction itself.

To address the challenges posed by objective assessments and the possibility that the shared situation‐level variances may not be substantial enough to be well explained by a covariate, it might be useful in future ESM studies to also assess students' perceived IC of a lecture. This could provide a more nuanced understanding of how subjectively perceived instructional clarity impacts both intra‐individual motivation in specific learning situations as well as dispositional motivation. If IC is added as a situation‐specific variable at Level 1 (ESM‐survey), the perceived clarity from the lecturer in a particular learning situation would be directly linked to an individual's momentary motivation. If IC is added at Level 2a (students) as a stable, average perception over the semester, it would explore how students' overall perception of IC relates to their dispositional motivation. The assumption here is that students who consistently perceive IC as higher are likely to develop a more stable or higher dispositional motivation. Additionally, examining the association between objective and subjective measures of instructional clarity would be an interesting avenue for future research, as our study could not provide such insights. It would clarify whether subjective perceptions of clarity systematically align with objective indicators or whether discrepancies exist, while also offering insights into the verbal and non‐verbal cues students use when forming their judgements and how the perceptions vary across different IC aspects.

### Strength, limitations and future directions

#### Strengths

First, the situational research design captures motivation in real time and in its natural setting in order to link it to the dynamics of teaching behaviour. This approach allows us to disentangle objective situational characteristics from students' subjective perceptions thereof (Moeller et al., 2020). Second, intensive ESM studies typically use single‐item measures to minimize participant burden. To address potential threats to reliability and validity, we assess motivational beliefs with multiple items (Dietrich et al., 2017; Moeller et al., 2020). Third, video recordings may distort normal teaching situations because lecturers and teachers might maximize their efforts and the variability of teaching behaviour can only be mapped to a limited extent (Clausen, 2002; Hiebert et al., 2003). Our study benefits from a lengthy observation period, which likely reduces these effects along with socially desirable behaviour.

#### Limitations

All models showed the smallest amount of variance at the learning situation‐level, which, in terms of an objective component, represents situation‐specific changes in the average motivation of all students from moment to moment. Transferred, this means John and Olivia share common (objective) motivation by experiencing the same lecture situation, but only to a small extent.

Furthermore, the participants were sampled from the same course in the same graduate program. Future studies should include multiple lectures with different lecturers to increase variance and improve the generalizability of the results.

There are some limitations regarding the observer ratings: First, we forewent a global rating of IC and formulated multiple items. This approximation to low‐inference coding may lose important aspects of validity, since in high‐inference rating systems deep structures are better captured and multiple aspects are integrated into a global judgement (Lotz, Gabriel, et al., 2013). Second, even though observer ratings are often used to objectively measure teaching quality, studies suggest that rater training does not necessarily improve rating quality, measures often contain a large amount of error variance and high reliability does not guarantee high validity (Praetorius et al., 2012). A major strength of objective ratings is their ability to exclude common method bias as the measurements of the predictor and criterion variables were obtained from different sources (Podsakoff et al., 2012). Nevertheless, the individual perception of the situation rather than objective situation characteristics is likely to be decisive for motivation. Therefore, it would be worthwhile to integrate the subjective perception of lecturer behaviour in the future. Moreover, averaging across three observation intervals before a beep reduces variance and might mask situational fluctuations.

Although this study included many situational observations, the limited number of measurement points at the learning situation level (*N* = 81) and the small, decreasing sample size of students (*N* = 155) reduced the power of the analyses on shared inter‐individual mean motivation of all students and intra‐individual motivational dispositions and may have led to selectivity bias. Furthermore, the systematic missingness in participation, influenced by performance and opportunity costs, may have resulted in an overrepresentation of more motivated students, potentially distorting the results.

#### Future research

Much more variance in motivation was driven by individual student characteristics than by the learning situation, suggesting that personal traits may play a key role in how students perceive and respond to IC. Future research should explore how these characteristics—such as prior knowledge or information processing—interact with situational cues. Initial evidence for temporal delays in cognitive processing that precede motivational appraisals warrants further attention, and it remains an open empirical question exactly how these processes take place and what cognitive mechanisms are involved. Understanding this could provide deeper insights into how instructional clarity influences learning motivation. The attendant challenge is to formulate appropriate theoretical models that account for these cognitive processes that precede motivational appraisals. Further knowledge about antecedents of expectancies and values at finer grain levels brings us a better understanding of what instructional techniques can be used effectively. At this point, it should be mentioned that experimental studies are needed to uncover causal dynamics between IC and motivation.

## CONCLUSION

The ebb and flow of expectancies and values has theoretical implications that highlight the activity‐, situation‐ and person‐dependent nature of motivational experiences. A key finding is the negation of the question of whether IC affects motivation in the same way for all students. In fact, the share of motivation that is equally shaped by the learning situation is in general low. This suggests that a student's motivation is less influenced by the objective features of the learning situation than we originally hypothesized. We conclude that a student's motivation in a situation is less influenced by the objective clarity of instruction – with some exceptions for the level of detail. The individual perception of IC rather than objective evaluation could be decisive for motivation.

## AUTHOR CONTRIBUTIONS

**Alina Oschwald:** Writing – original draft; formal analysis. **Julia Moeller:** Writing – review and editing; investigation. **Bärbel Kracke:** Writing – review and editing. **Jaana Viljaranta:** Writing – review and editing; investigation. **Julia Dietrich:** Conceptualization; methodology; investigation; writing – review and editing; supervision; project administration.

## CONFLICT OF INTEREST STATEMENT

The authors declare no conflicts of interest.

## Supporting information


Appendix S1:

Appendix S2:

## Data Availability

The data that support the findings will be available on OSF at https://osf.io/xgjhz/?view_only=5c47f5068bca47a8907ef174e6fd2b26 from the date of publication.
