# Supplementary material for: Student motivation and instructional clarity: Linking experience sampling method data to objective behavioural observations
Source: Br J Educ Psychol. 2025 Apr 18;95(Suppl 1):S281–99. doi: 10.1111/bjep.12775 (PMC12427158; doi:10.1111/bjep.12775)
Supplement: Supplementary file 1 — Appendix S1: [file BJEP-95-S281-s001.docx]

**Appendices – Online Supplements**

Supplementary data related to this article can be found at <https://osf.io/xgjhz/?view_only=fcfaebbf2fb94d0d8a8e1ef6777deb55>
