# Supplementary material for: Student motivation and instructional clarity: Linking experience sampling method data to objective behavioural observations
Source: Br J Educ Psychol. 2025 Apr 18;95(Suppl 1):S281–99. doi: 10.1111/bjep.12775 (PMC12427158; doi:10.1111/bjep.12775)
Supplement: Supplementary file 2 — Appendix S2: [file BJEP-95-S281-s002.zip › Appendix A - Student Motivation/Appendix A - Student Motivation.pdf]

Appendix A – Motivation

A.1 Measures

Table A.1 *Items of the situational motivation questionnaire*

Variable Name	Label (English)	Label (German)
	**Instruction (English)**<br>The following questions pertain to the lecture contents of the last couple of minutes which were talked about shortly before you received the beep signal.<br><br>Please fill in within 10 minutes after the signal.<br><br>To what extent do the following statements apply to you in the present moment?	**Instruction (German)**<br>Die folgenden Fragen beziehen sich auf die Vorlesungsinhalte der letzten Minuten, die besprochen wurden, kurz bevor Sie das Signal zum Fragebogen bekamen.<br>Bitte füllen Sie innerhalb von 10 Minuten nach dem Signal aus.<br><br>Wie sehr treffen folgende Aussagen jetzt gerade auf Sie zu?
Situational expectancies		
Expectation of success	I will do well in the exam covering these contents.	Diese Inhalte werde ich in der Klausur gut können.
Competence beliefs	I understand these contents.	Ich verstehe diese Inhalte.
Situational task values		
Intrinsic value	I like these contents.	Ich mag diese Inhalte.
Attainment value	It is important for me to know a lot about these contents.	Mir ist es wichtig, über diese Inhalte viel zu wissen.
Utility value	Knowing these contents well will be useful in my future occupation.	Diese Inhalte werden für meinen späteren Beruf nützlich sein.
Effort costs	Learning these contents exhausts me.	Diesen Inhalten zu folgen, erschöpft mich.
Emotional costs	I feel bad because I need to deal with these contents (e.g., I am annoyed and/or anxious and/or nervous).	Ich fühle mich schlecht, weil ich mich mit diesen Inhalten beschäftigen muss (z.B. ich bin genervt, und/oder ängstlich, und/oder nervös).
Opportunity costs	I have to give up other activities that I like in order to know these contents well.	Um mich mit diesen Inhalten gut auszukennen, muss ich andere Aktivitäten aufgeben, die mir Spaß machen.

Note. Response format for motivation measures (1 = *does not apply*, 2 = *rather not applies*, 3 = *rather applies*, 4 = *fully applies*). Original German response format (1 = *stimmt gar nicht*, 2 = *stimmt eher nicht*, 3 = *stimmt eher*, 4 = *stimmt eher nicht*).

A.2 Descriptive Results

Table A.2.1 displays the descriptive statistics for students' motivation separated by lectures (*N* = 9) and Table A.2.2 for each of the 81 measurement time points. On average, students reported high competence beliefs (range of mean motivation and standard deviation among all students between measurement time points *M* = 2.71 – 3.63; *SD* = .37 – .83) and moderate to high expectations of success (*M* = 2.43 – 3.37; *SD* = .23 – .95). The utility of the content of the course for future occupation was rated highest by the students (*M* = 2.57 – 3.55; *SD* = .39 – 1.01), and moderate to high ratings were given for intrinsic (*M* = 2.25 – 3.29; *SD* = .39 – .94) and attainment value (*M* = 2.25 – 3.22; *SD* = .46 – 1.03). Low to medium values were reported on average for each of the three cost components, lowest for emotional costs (*M* = 1.00 – 2.00; *SD* = .00 – 1.03) followed by opportunity costs (*M* = 1. 00 – 2.14; *SD* = .00 – 1.35) and effort costs (*M* = 1.11 – 2.40; *SD* = .33 – 1.28). With regard to the average motivation of all students across a lecture, the overall assessment of motivation was high with differences between sessions (see Table A.2.1). The highest motivational responses occurred for the first lecture (topic: adolescent development), the lowest for the last lecture (topic: diagnostics and evaluation). The standard deviations varied widely within measurement time points, such that students' motivation was more similar in some situations and less similar in others. Considering the standard deviations across lectures (see Table A.2.1), there were more often high standard deviations in opportunity costs and effort costs compared to expectations and values. Overall, these results indicated considerable differences in motivation between measurement time points, sessions and within individuals.

Table A.2.1 *Descriptive item statistics separate for lectures*

Lecture Topic	Item	Intervall	N_1	n_2	*M*	*SD*
1. Adolescent development	Situational expectancies	Expectations of success	151	374	3.01	0.52
		Competence beliefs	151	367	3.40	0.54
	Situational task values	Intrinsic value	151	374	3.11	0.63
		Attainment value	151	373	3.02	0.64
		Utility value	151	375	3.23	0.65
		Effort Costs	151	374	1.80	0.75
		Emotional Costs	151	371	1.43	0.59
		Opportunity Costs	151	376	1.60	0.69
2. Knowledge acquisition	Situational expectancies	Expectations of success	124	329	2.90	0.56
		Competence beliefs	124	326	3.11	0.58
	Situational task values	Intrinsic value	124	329	2.98	0.66
		Attainment value	124	329	2.88	0.69
		Utility value	124	328	3.14	0.66
		Effort Costs	124	327	2.16	0.90
		Emotional Costs	124	328	1.66	0.72
		Opportunity Costs	124	328	1.74	0.79
3. Intelligence and prior knowledge	Situational expectancies	Expectations of success	105	261	2.94	0.65
		Competence beliefs	105	260	3.30	0.58
	Situational task values	Intrinsic value	105	261	2.98	0.66
		Attainment value	105	261	2.92	0.66
		Utility value	105	259	3.02	0.71
		Effort Costs	105	261	1.97	0.84
		Emotional Costs	105	261	1.57	0.69
		Opportunity Costs	105	261	1.74	0.79
5. Motivation	Situational expectancies	Expectations of success	84	213	3.05	0.54
		Competence beliefs	84	209	3.25	0.56
	Situational task values	Intrinsic value	84	213	3.00	0.52
		Attainment value	84	212	2.90	0.60
		Utility value	84	212	3.25	0.59
		Effort Costs	84	212	1.85	0.79
		Emotional Costs	84	211	1.48	0.66
		Opportunity Costs	84	211	1.71	0.83
6. Self-concept	Situational expectancies	Expectations of success	89	208	2.99	0.54
		Competence beliefs	89	207	3.18	0.55
	Situational task values	Intrinsic value	89	208	2.86	0.68
		Attainment value	89	208	2.78	0.64
		Utility value	89	207	3.07	0.60
		Effort Costs	89	207	1.98	0.85
		Emotional Costs	89	208	1.62	0.71
		Opportunity Costs	89	208	1.71	0.83

Lecture (Topic)	Item	Intervall	*N₁*	*n₂*	*M*	*SD*
7. Emotion	Situational expectancies	Expectations of success	80	172	3.06	0.54
		Competence beliefs	80	170	3.29	0.53
	Situational task values	Intrinsic value	80	172	2.96	0.62
		Attainment value	80	172	2.92	0.68
		Utility value	80	172	3.16	0.59
		Effort Costs	80	172	1.95	0.90
		Emotional Costs	80	172	1.59	0.76
		Opportunity Costs	80	172	1.60	0.83
8. Family & peer context	Situational expectancies	Expectations of success	70	149	2.99	0.60
		Competence beliefs	70	149	3.21	0.68
	Situational task values	Intrinsic value	70	149	2.91	0.68
		Attainment value	70	149	2.85	0.67
		Utility value	70	148	3.11	0.65
		Effort Costs	70	148	1.85	0.79
		Emotional Costs	70	149	1.51	0.68
		Opportunity Costs	70	149	1.89	0.93
9. School context	Situational expectancies	Expectations of success	69	158	3.17	0.43
		Competence beliefs	69	156	3.34	0.50
	Situational task values	Intrinsic value	69	158	3.14	0.67
		Attainment value	69	158	3.01	0.67
		Utility value	69	157	3.25	0.58
		Effort Costs	69	155	1.79	0.73
		Emotional Costs	69	157	1.51	0.68
		Opportunity Costs	69	158	1.64	0.81
10. Diagnostics and evaluation	Situational expectancies	Expectations of success	61	144	2.95	0.60
		Competence beliefs	61	144	3.17	0.61
	Situational task values	Intrinsic value	61	144	2.62	0.67
		Attainment value	61	144	2.72	0.69
		Utility value	61	143	2.98	0.64
		Effort Costs	61	144	1.98	0.81
		Emotional Costs	61	144	1.69	0.72
		Opportunity Costs	61	144	1.78	0.89
All lectures	Situational expectancies	Expectations of success	155	144	2.95	0.60
		Competence beliefs	155	144	3.17	0.61
	Situational task values	Intrinsic value	155	2,218	2.98	0.57
		Attainment value	155	2,198	3.24	0.59
		Utility value	155	2,220	2.91	0.66
		Effort Costs	155	2,217	2.88	0.67
		Emotional Costs	155	2,212	3.13	0.64
		Opportunity Costs	155	2,211	1.95	0.83

Note. N_1 = participants, n_2 = motivational responses, M = Mean, SD = Standard deviation, MSSD = mean square successive difference, r = autocorrelation

A.3 Results of the unconditional cross-classified multilevel model (M0)

We computed an unconditional cross-classified multilevel model to decompose variance of students' situational expectancies, values and costs at the situational level (within, *N* = 2227), at the person-specific level (between students, *N* = 155), as well as at the time point-specific level (between time points, *N* = 81). The composite SEVT components correspond to those from the main analyses of research question 2. See Table A.3.1 for variance component estimates and Table A.3.2 for item intercorrelations at all levels. For this more complex model, compared to single motivation items, a thinning of 10 was set. The *ppp*-value was .455 and the 95% CI for the difference between observed and replicated chi-squared values comprises zero [-20.110; 21.686], indicating an excellent model fit (Muthén et al., 2012).

Table A.3.1 *Unconditional cross-classified multilevel model (M0).*

	Estimates [*CI*]
Means	
Expectancies	3.099
Task values	2.991
Costs	1.792
Variances	
L1 Expectancies	.166 [.155; .177]
L1 Task values	.177 [.166; .189]
L1 Costs	.166 [.156; .178]
L2a Expectancies	.089 [.066; .120]
L2a Task values	.130 [.100; .172]
L2a Costs	.240 [.186; .312]
L2b Expectancies	.010 [.005; .017]
L2b Task values	.014 [.008; .023]
L2b Costs	.013 [.008; .022]
ICC	
L2a Expectancies	.336 [.272; .407]
L2a Task values	.405 [.340; .476]
L2a Costs	.571 [.506; .638]
L2b Expectancies	.037 [.020; .062]
L2b Task values	.043 [.025; .070]
L2b Costs	.032 [.019; .051]

Note. CI = Credibility Interval.

Table A.3.2 *Item intercorrelations on within-level ((level 1: motivational responses) and between-levels (level 2a: students and level 2b: time points) from the unconditional cross-classified multilevel model (M0).*

	Situational expectancies	Situational task values	Situational costs
**Level 1 (within motivational responses)**			
Situational expectancies	–		
Situational task values	.51	–	
Situational costs	-.27	-.30	–
**Level 2a (between students)**			
Situational expectancies	–		
Situational task values	.58	–	
Situational costs	-.46	-.30	–
**Level 2b (between time points)**			
Situational expectancies	–		
Situational task values	.86	–	
Situational costs	-.88	-.87	–

Note. All credibility intervals do not include 0.

A.4 Results of the unconditional cross-classified multilevel model (M0.a)

We computed another unconditional cross-classified multilevel model (M0.a) to examine the structure of item-intercorrelations of each facet of the SEVT components (e.g., intrinsic value, attainment value, utility value, etc.) at the response-level (within, *N* = 2227), at the person-specific level (between students, *N* = 155), as well as at the time point-specific level (between time points, *N* = 81). See Table A.4.1 for variance component estimates and Table A.4.2 for item intercorrelations at all levels. For this more complex model, which included all single motivational items, a thinning of 10 was set. The model fit was acceptable (*ppp-value* = .250) with lower and upper limits of the 95% confidence interval that included zero [-30.005; 66.190].

The results in Table A.4.2 show positive correlations within expectations, (L1: *r* = .46, L2a: *r* = .62, L2b: *r* = .55), within values (L1: *r* = .39 to .49, L2a: *r* = .68 to .81, L2b: *r* = .67 to .83), and within costs values (L1: *r* = .30 to .33, L2a: *r* = .60 to .79, L2b: *r* = .52 to .70) on all levels. Correlations were higher at student-level and time point-level compared to response-level. As expected, expectations and values were positively associated (L1: *r* = .27 to .44, L2a: *r* = .43 to .54, L2b: *r* = .50 to .83), while costs were negatively associated with expectations (L1: *r* = -.11 to -.20, L2a: *r* = -.25 to -.45, L2b: *r* = -.45 to -.79) and values (L1: *r* = -.11 to -.25, L2a: *r* = -.33 to -.45, L2b: *r* = -.41 to -.85). This correlation structure was found at all levels with exceptions for opportunity cost; correlations at the time point-level were highest.

Table A.4.1 *Unconditional cross-classified multilevel model (M0a).*

	Estimates [*CI*]
Means	
Expectations of success	2.985
Competence beliefs	3.226
Intrinsic value	2.934
Attainment value	2.905
Utility value	3.126
Effort Costs	1.971
Emotional Costs	1.627
Opportunity Costs	1.786
Variances	
L1 Expectations of success	.221 [.207; .236]
L1 Competence beliefs	.230 [.215; .245]
L1 Intrinsic value	.305 [.285; .325]
L1 Attainment value	.273 [.256; .292]
L1 Utility value	.270 [.253; .289]
L1 Effort Costs	.368 [.344; .393]
L1 Emotional Costs	.283 [.266; .303]
L1 Opportunity Costs	.266 [.249; .285]
L2a Expectations of success	.114 [.085; .155]
L2a Competence beliefs	.123 [.090; .169]
L2a Intrinsic value	.136 [.100; .184]
L2a Attainment value	.211 [.160; .282]
L2a Utility value	.175 [.131; .234]
L2a Effort Costs	.349 [.266; .461]
L2a Emotional Costs	.228 [.173; .303]
L2a Opportunity Costs	.461 [.358; .608]
L2b Expectations of success	.011 [.006; .021]
L2b Competence beliefs	.023 [.014; .037]
L2b Intrinsic value	.030 [.017; .049]
L2b Attainment value	.016 [.008; .029]
L2b Utility value	.020 [.011; .035]
L2b Effort Costs	.034 [.020; .057]
L2b Emotional Costs	.019 [.011; .034]
L2b Opportunity Costs	.016 [.009; .029]
ICC	
L2a Expectations of success	.329 [.264; .402]
L2a Competence beliefs	.327 [.262; .403]
L2a Intrinsic value	.288 [.227; .358]
L2a Attainment value	.421 [.351; .496]
L2a Utility value	.375 [.307; .449]
L2a Effort Costs	.464 [.395; .536]
L2a Emotional Costs	.429 [.359; .501]
L2a Opportunity Costs	.620 [.556; .684]
L2b Expectations of success	.033 [.017; .059]
L2b Competence beliefs	.061 [.037; .097]
L2b Intrinsic value	.063 [.038; .101]
L2b Attainment value	.032 [.017; .057]
L2b Utility value	.043 [.024; .074]
L2b Effort Costs	.045 [.027; .075]

L2b Emotional Costs	.037 [.020; .062]
L2b Opportunity Costs	.022 [.012; .039]

Note. CI = Credibility Interval.

Table A.4.2 *Unconditional cross-classified multilevel model (M0a). Item intercorrelations on within-level (level 1: motivational responses) and between-levels (level 2a: students and level 2b: time points).*

	1	2	3	4	5	6	7	8
**Level 1 (within motivational responses)**								
Situational expectancies								
1 Expectation of success	–							
2 Competence beliefs	.46	–						
Situational task values								
3 Intrinsic value	.44	.39	–					
4 Attainment value	.38	.31	.49	–				
5 Utility value	.33	.27	.39	.45	–			
6 Effort costs	-.20	-.19	-.21	-.21	-.12	–		
7 Emotional costs	-.20	-.20	-.25	-.19	-.16	.33	–	
8 Opportunity costs	-.13	-.11	-.17	-.17	-.11	.30	.30	–
**Level 2a (between students)**								
Situational expectancies								
1 Expectation of success	–							
2 Competence beliefs	.62	–						
Situational task values								
3 Intrinsic value	.43	.48	–					
4 Attainment value	.49	.43	.81	–				
5 Utility value	.54	.46	.68	.75	–			
6 Effort costs	-.43	-.45	-.42	-.33	-.36	–		
7 Emotional costs	-.26	-.41	-.45	-.36	-.37	.79	–	
8 Opportunity costs	-.25	-.36	-.11[1]	.03[1]	-.07[1]	.64	.60	–
**Level 2b (between time points)**								
Situational expectancies								
1 Expectation of success	–							
2 Competence beliefs	.55	–						
Situational task values								
3 Intrinsic value	.62	.83	–					
4 Attainment value	.61	.66	.83	–				
5 Utility value	.62	.50	.67	.69	–			
6 Effort costs	-.63	-.76	-.67	-.65	-.41	–		
7 Emotional costs	-.45	-.79	-.85	-.72	-.48	.69	–	
8 Opportunity costs	-.21[1]	-.64	-.73	-.71	-.41	.52	.70	–

Note. [1] Credibility intervals include 0.

References

Muthén, B., & Asparouhov, T. (2012). Bayesian structural equation modeling: a more flexible representation of substantive theory. *Psychological Methods*, *17*(3), 313-335. https://doi.org/10.1037/a0026802
