# Supplementary material for: Student motivation and instructional clarity: Linking experience sampling method data to objective behavioural observations
Source: Br J Educ Psychol. 2025 Apr 18;95(Suppl 1):S281–99. doi: 10.1111/bjep.12775 (PMC12427158; doi:10.1111/bjep.12775)
Supplement: Supplementary file 2 — Appendix S2: [file BJEP-95-S281-s002.zip › Appendix B - Instructional Clarity/Appendix B - Instructional Clarity.pdf]

Appendix B – Instructional Clarity

B.1 Measures

Table B.1 *Operationalization of instructional clarity*

Operationalization (English)	Operationalization (German)
Positive Indicators	
• The teacher explained content in detail (explanation in depth).	• Die Lehrperson erklärt Inhalte detailliert (Erklärungstiefe).
• The teacher explained content in different variations (explanation in width). Several examples can also be diverse e.g., alternating between analogy, metaphor, example and explanation.	• Die Lehrperson erklärt Inhalte in verschiedenen Variationen (Erklärungsbreite). Variantenreich können auch mehrere Beispiele sein, z. B. abwechselnd Analogie, Metapher, Beispiel und Erklärung.
Negative Indicator	
• The teacher's argumentation shows gaps and is not consistent and stringent in its logic. Digressing from the topic can be part of that, but only if it interferes with the logic of the argument. The logic of argumentation can refer to an abstract content or to an example. This is about the structure of the argumentation.	• Die Lehrperson springt in ihrer Argumentationslogik und diese weist Lücken auf (keine stringente Argumentation, Abschweifen). Abschweifen vom Thema zählt auch dazu, jedoch nur dann, wenn Argumentationslogik unterbrochen wird. Die Argumentation kann sich auf einen abstrakten Inhalt oder ein Beispiel beziehen. Hier geht es um die Struktur der Argumentation.

All three indicators were rated in each observational unit, with exactly one code assigned to each indicator. For this purpose, a three-point scale was used (0 = *not/never observed*, 1 = *partly observed*, 2 = *continuously observed* in the three minutes)[1]. By 'not/never observed', it meant that

[1] Original German response format (1 = *nie/nicht beobachtet*, 2 = *teilweise beobachtet*, 3 = *durchgehend beobachtet*). Mit "nicht/nie beobachtet" ist gemeint, dass der Indikator zu keinem Zeitpunkt des Intervalls erfüllt war. Mit "teilweise beobachtet" ist gemeint, dass der Indikator teilweise, d. h. punktuell, für eine Minute oder weniger, auftrat. Wenn ein Indikator zwei Minuten oder länger innerhalb eines Intervalls auftritt, wurde der Code "durchgehend beobachtet" vergeben. Viele kurze oder undetaillierte Beispiele werden als "teilweise beobachtet" anstelle von "durchgehend beobachtet" zusammengefasst.

the indicator had not been fulfilled at any time in the interval. By ‘partly observed’, it meant that the indicator occurred partly, i.e., for one minute or less. If an indicator is occurring for two minutes or more within an interval, the code ‘continuously observed’ was assigned. Many short or undetailed examples are summarized as ‘partly observed’ instead of ‘continuously observed’.

The videos were analyzed not only for instructional clarity but also regarding other constructs, including practical and real-world relevance, structural cues within the lecture, and linguistic comprehensibility. Due to low inter-rater reliability, no further analyses were conducted with this data.

B.2 Descriptive Results

Table B.2.1 presents descriptive statistics of instructional clarity for each of the nine weeks, Table B.2.2 shows the results separated by intervals (n = 3) and Table B.2.3 for each of the 81 measurement time points. During the lectures (Table B.2.1), the lecturer explained the content mainly in detail (range of mean instructional clarity and standard deviations between weeks M = 0.63 – 1.46, SD = .50 – .74), with less variation (M = 0.50 – 1.00; SD = .50 – .75). In all sessions, the lecturer's argumentation was consistent in its logic (M = 0.04 – 0.47; SD = .20 – .49). Overall, these results suggest that instructional clarity was high over the course of the lecture, with greater variability in detail of explanation and variation of explanation between sessions. As intended, there were no differences in observations between the intervals (similar means and standard deviations, see Table B.2.2). Table B.2.3 presents the means and the standard deviations of the video observations for each of the 81 measurement time points (three observations prior to a beep were averaged). For detail of explanation (range of mean instructional clarity and standard deviations between measurement time points M = 0.00 – 2.00; SD = .00 – 1.41) and for variation of explanation (M = 0.00 – 2.00; SD = .00 – 1.41), there were moments in which the indicator was never observed and moments in which the indicator was consistently observed. Across all measurement time points, the lecturer's argumentation was never continuously inconsistent in its logic (M = 0.00 – 1.00; SD = .00 – 1.15).

Table B.2.1 *Descriptive item statistics separate for lectures*

Item	Lecture / week	*n*	*M*	*SD*	*MSSD*	*r*	Absolute and Relative Frequencies		
							0 = *not/never observed*	1 = *partly observed*	2 = *continuously observed*
Detail	1	27	1.15	0.72	0.85	.09[a]	5 (18.52%)	13 (48.15%)	9 (33.33%)
	2	27	0.63	0.56	0.58	.07[a]	11 (40.74%)	15 (55.56%)	1 (3.70%)
	3	27	0.81	0.74	0.92	.10[a]	10 (37.04%)	12 (44.44%)	5 (18.52%)
	5	27	0.93	0.68	1.08	-.18[a]	7 (25.93%)	15 (55.56%)	5 (18.52%)
	6	26	1.46	0.65	0.58	.30[a]	2 (7.41%)	10 (37.04%)	14 (51.85%)
	7	25	1.12	0.60	0.46	.32[a]	3 (11.11%)	16 (59.26%)	6 (22.22%)
	8	26	1.04	0.66	0.96	-.22[a]	5 (18.52%)	15 (55.56%)	6 (22.22%)
	9	26	1.27	0.67	0.96	-.27[a]	3 (11.11%)	13 (48.15%)	10 (37.04%)
	10	27	1.41	0.50	0.31	.19[a]	-	16 (59.26%)	11 (40.74%)
Variation	1	27	0.70	0.57	0.35	.15[a]	8 (29.63%)	19 (70.37%)	-
	2	27	0.70	0.67	0.58	.33[a]	11 (40.47%)	13 (48.15%)	3 (11.11%)
	3	27	0.67	0.73	0.35	.67	13 (48.15%)	10 (37.04%)	4 (14.81%)
	5	27	0.78	0.75	1.00	.07[a]	11 (40.74%)	11 (40.74%)	5 (18.52%)
	6	26	0.65	0.56	0.75	-.29[a]	10 (37.04%)	15 (55.56%)	1 (3.70%)
	7	25	1.00	0.50	0.42	-.01[a]	3 (11.11%)	19 (70.37%)	3 (11.11%)
	8	26	0.50	0.65	0.88	-.07[a]	15 (55.56%)	9 (33.33%)	2 (7.41%)
	9	26	0.58	0.50	0.32	.33[a]	11 (40.74%)	15 (55.56%)	-
	10	27	0.96	0.59	0.42	.34[a]	5 (18.52%)	18 (66.56%)	4 (14.81%)
Logical Incon-sistency	1	27	0.11	0.32	0.12	.29[a]	24 (88.89%)	3 (11.11%)	-
	2	27	0.15	0.36	0.31	-.18[a]	23 (85.19%)	4 (14.81%)	-
	3	27	0.37	0.49	0.38	.19[a]	17 (62.96%)	10 (37.04%)	-
	5	27	0.22	0.42	0.46	-.30[a]	21 (77.78%)	6 (22.22%)	-
	6	26	0.04	0.20	0.08	-.04[a]	25 (92.59%)	1 (3.70%)	-
	7	25	0.04	0.20	0.08	-.04[a]	24 (88.89%)	1 (3.70%)	-
	8	26	0.42	0.50	0.36	.26[a]	15 (55.56%)	11 (40.74%)	-
	9	26	0.19	0.49	0.40	.17[a]	22 (81.48%)	3 (11.11%)	1 (3.70%)
	10	27	0.07	0.27	0.15	-.08[a]	25 (92.59%)	2 (7.41%)	-

Note. n = video observations, M = Mean, SD = Standard deviation, MSSD = mean square successive difference, r = autocorrelation.
[a] = not significant

Table B.2.2 *Descriptive item statistics separate for intervals*

Item	Intervall	*n*	*M*	*SD*	Absolute and Relative Frequencies		
					0 = *not/never observed*	1 = *partly observed*	2 = *continuously observed*
Detail	1	78	1.09	0.71	16 (19.75%)	39 (48.15%)	23 (28.40%)
	2	80	1.05	0.63	14 (17.28%)	48 (59.26%)	18 (22.22%)
	3	80	1.12	0.72	16 (19.75%)	38 (46.91%)	26 (32.10%)
	**all intervals**	**238**	**1.09**	**0.68**	**46 (18.93%)**	**125 (51.44%)**	**67 (27.57%)**
Variation	1	78	0.71	0.63	30 (37.04%)	41 (50.62%)	7 (8.64%)
	2	80	0.73	0.59	28 (34.57%)	46 (56.79%)	6 (7.41%)
	3	80	0.75	0.65	29 (35.80%)	42 (51.85%)	9 (11.11%)
	**all intervals**	**238**	**0.73**	**0.62**	**87 (35.80%)**	**129 (53.09%)**	**22 (9.05%)**
Logical Inconsistency	1	78	0.22	0.45	62 (76.54%)	15 (18.52%)	1 (1.23%)
	2	80	0.21	0.41	63 (77.78%)	17 (20.99%)	-
	3	80	0.11	0.32	71 (87.65%)	9 (11.11%)	-
	**all intervals**	**238**	**0.18**	**0.40**	**196 (80.66%)**	**41 (16.87%)**	**1 (0.41%)**

Note. n = video observations, M = Mean, SD = Standard deviation.

Table B.2.3 *Descriptive item statistics separate for measurement time points*

Time point	Detail		Variation		Logical Inconsistency	
	M	*SD*	*M*	*SD*	*M*	*SD*
**11**	0.67	1.15	0.33	0.58	0.33	0.58
**12**	1.00	0.00	0.33	0.58	0.00	0.00
**13**	1.33	0.58	0.67	0.58	0.00	0.00
**14**	1.33	0.58	1.00	0.00	0.00	0.00
**15**	1.33	0.58	0.67	0.58	0.00	0.00
**16**	1.00	1.00	1.00	0.00	0.00	0.00
**17**	1.67	0.58	0.67	0.58	0.67	0.58
**18**	1.67	0.58	0.67	0.58	0.00	0.00
**19**	0.33	0.58	1.00	0.00	0.00	0.00
**21**	0.33	0.58	0.33	0.58	0.00	0.00
**22**	0.67	0.58	0.33	0.58	0.00	0.00
**23**	0.67	0.58	0.00	0.00	0.67	0.58
**24**	0.67	0.58	1.67	0.58	0.00	0.00
**25**	0.67	0.58	1.33	0.58	0.00	0.00
**26**	0.33	0.58	0.67	0.58	0.33	0.58
**27**	0.33	0.58	0.67	0.58	0.00	0.00
**28**	1.33	0.58	0.33	0.58	0.00	0.00
**29**	0.67	0.58	1.00	0.00	0.33	0.58
**31**	0.00	0.00	0.00	0.00	0.00	0.00
**32**	0.33	0.58	0.33	0.58	0.33	0.58
**33**	1.00	1.00	1.33	0.58	0.00	0.00
**34**	0.67	0.58	0.00	0.00	0.33	0.58
**35**	1.33	0.58	1.00	0.00	1.00	0.00
**36**	1.67	0.58	0.00	0.00	0.33	0.58
**37**	1.00	1.00	0.33	0.58	0.67	0.58
**38**	1.00	0.00	2.00	0.00	0.00	0.00
**39**	0.33	0.58	1.00	0.00	0.67	0.58
**51**	0.67	0.58	0.00	0.00	0.33	0.58
**52**	1.33	0.58	1.33	0.58	0.00	0.00
**53**	1.00	1.00	1.33	0.58	0.33	0.58
**54**	1.33	1.15	0.67	0.58	0.00	0.00
**55**	1.00	1.00	0.67	1.15	0.33	0.58
**56**	1.00	0.00	0.33	0.58	0.33	0.58
**57**	0.67	0.58	0.67	0.58	0.33	0.58
**58**	0.67	0.58	1.00	1.00	0.00	0.00
**59**	0.67	0.58	1.00	1.00	0.33	0.58
**61**	1.67	0.58	0.67	0.58	0.00	0.00
**62**	1.00	0.00	0.67	0.58	0.00	0.00
**63**	1.00	1.00	0.67	0.58	0.00	0.00
**64**	0.67	0.58	1.00	1.00	0.33	0.58
**65**	1.33	0.58	0.67	0.58	0.00	0.00

**66**	2.00	0.00	0.50	0.71	0.00	0.00
**67**	1.67	0.58	0.67	0.58	0.00	0.00
**68**	2.00	0.00	0.67	0.58	0.00	0.00
**69**	2.00	0.00	0.33	0.58	0.00	0.00
**71**	2.00	-	0.00	-	0.00	-
**72**	1.67	0.58	1.00	0.00	0.33	0.58
**73**	1.33	0.58	1.00	0.00	0.00	0.00
**74**	1.00	0.00	1.00	0.00	0.00	0.00
**75**	0.33	0.58	1.33	1.15	0.00	0.00
**76**	0.67	0.58	1.00	0.00	0.00	0.00
**77**	1.33	0.58	1.33	0.58	0.00	0.00
**78**	1.33	0.58	1.00	0.00	0.00	0.00
**79**	1.00	0.00	0.67	0.58	0.00	0.00
**81**	1.00	1.41	1.00	1.41	0.50	0.71
**82**	1.33	0.58	1.00	0.00	0.67	0.58
**83**	1.00	1.00	0.33	0.58	0.33	0.58
**84**	0.67	0.58	0.00	0.00	0.33	0.58
**85**	1.00	1.00	0.67	0.58	1.00	0.00
**86**	1.33	0.58	1.00	1.00	0.00	0.00
**87**	1.33	0.58	0.00	0.00	0.00	0.00
**88**	1.00	0.00	0.33	0.58	0.33	0.58
**89**	0.67	0.58	0.33	0.58	0.67	0.58
**91**	0.33	0.58	0.00	0.00	0.33	0.58
**92**	1.33	0.58	0.33	0.58	0.67	1.15
**93**	1.33	0.58	0.33	0.58	0.00	0.00
**94**	1.33	0.58	0.67	0.58	0.00	0.00
**95**	1.67	0.58	0.67	0.58	0.00	0.00
**96**	1.33	0.58	1.00	0.00	0.33	0.58
**97**	1.33	0.58	1.00	0.00	0.00	0.00
**98**	1.67	0.58	1.00	0.00	0.33	0.58
**99**	1.00	1.41	0.00	0.00	0.00	0.00
**101**	1.00	0.00	0.67	0.58	0.33	0.58
**102**	1.33	0.58	1.33	0.58	0.00	0.00
**103**	1.67	0.58	0.33	0.58	0.00	0.00
**104**	1.67	0.58	1.00	0.00	0.00	0.00
**105**	2.00	0.00	0.67	0.58	0.00	0.00
**106**	1.67	0.58	0.67	0.58	0.00	0.00
**107**	1.00	0.00	1.00	0.00	0.33	0.58
**108**	1.33	0.58	1.67	0.58	0.00	0.00
**109**	1.00	0.00	1.33	0.58	0.00	0.00

Note. M = Mean. SD = standard deviation. Time point = first digit denotes the week, second digit the timing of the beep within a lecture.

B.3 Time series of instructional clarity

Figure B.3.1 Detail of Explanation

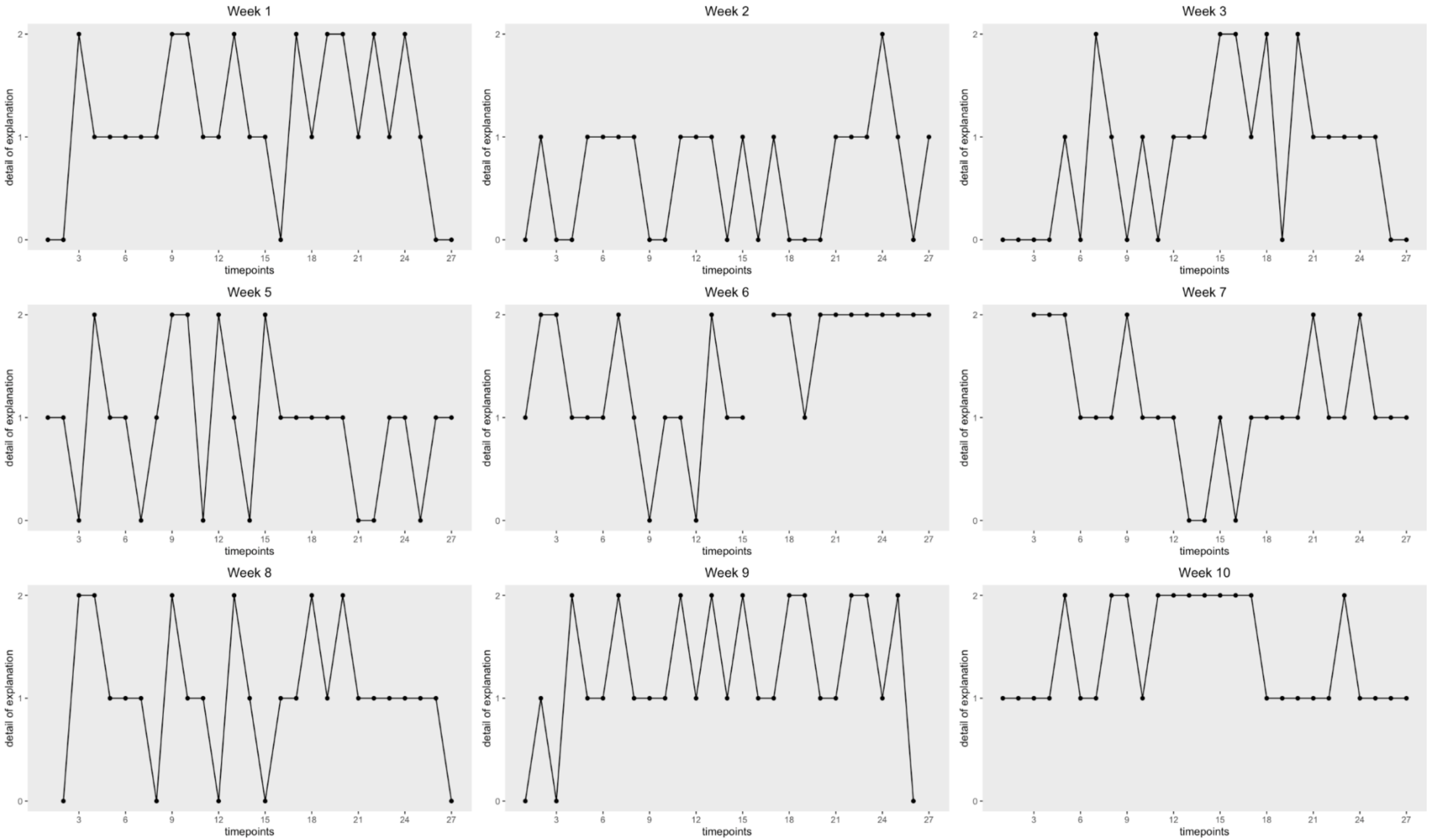

Figure B.3.2 Variation of Explanation

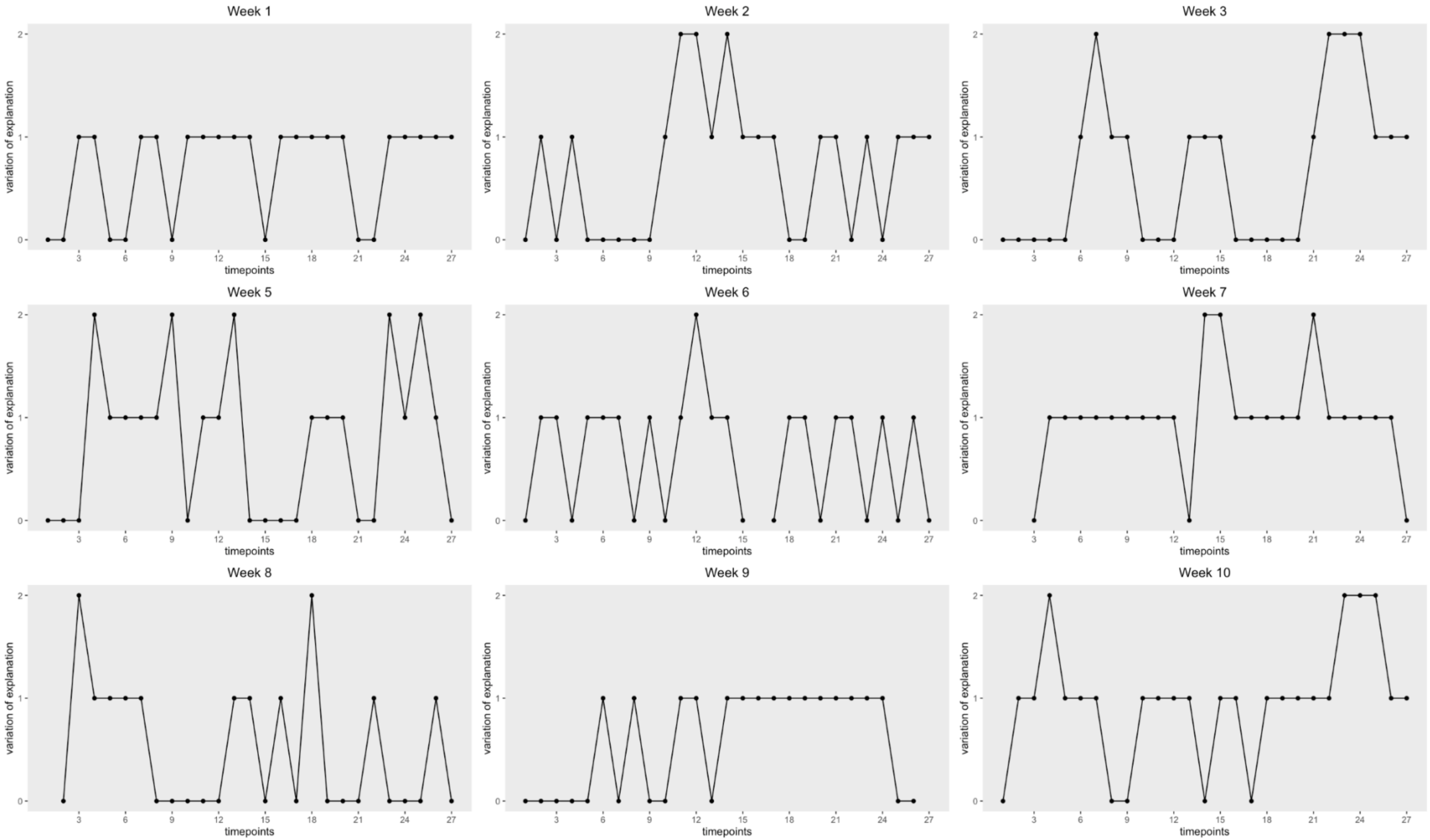

Figure B.3.3 Logical Inconsistency

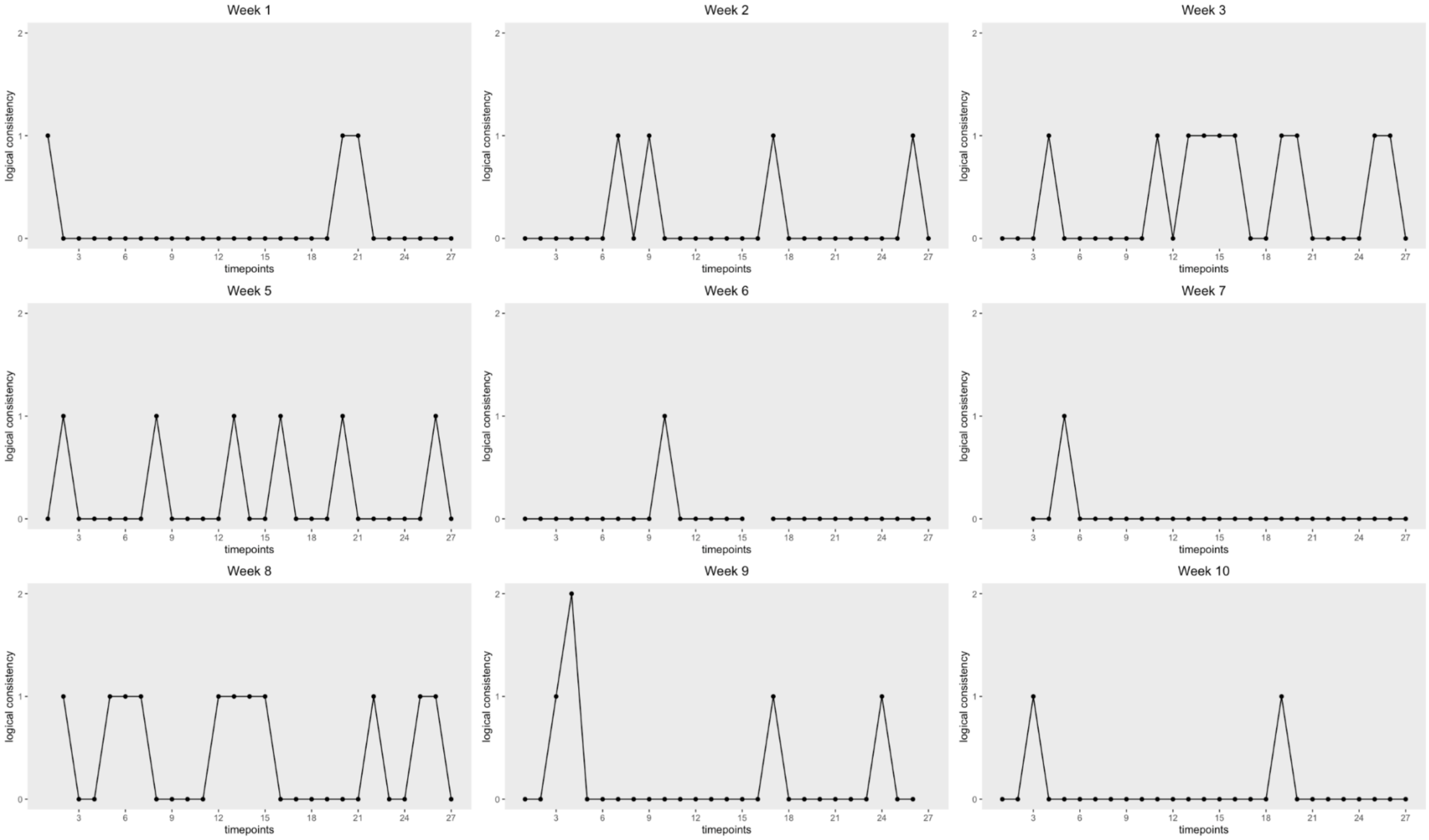

B.4 Interrater reliability

All intervals were rated independently by two raters who previously completed a training. Based on the independent ratings, the interrater reliability was calculated using several measures. All indicators have reached the requirements for sufficient data quality (confidence interval of ICCs ranged from .53 to .75; relative generalizability coefficient ranged from 0.76 to 0.82). In case of discrepancies, a consensus of the coding was generated afterwards (detail: for 57 of the 238 intervals (23.59%); variation: for 59 of the 238 intervals (24.79%); logical inconsistency: for 25 of the 238 intervals (10.50%)). For the analyses, the consensus codes were used.

We used a two-way mixed, single score ICC (C,1) because each case was assessed by two fixed raters and the data basis were raw rating scores from these single raters. According to the interpretation of Koo et al. (2016), ICCs between .5 and .75 indicate moderate reliability, ICCs between .75 and .9 indicate good reliability, and ICCs greater than .90 indicate excellent reliability. The ICCs confidence intervals for the three indicators were all within the range of moderate reliability (.53-.75).

The relative generalization coefficient is capable to reflect the potential sources of variance of a measured variation (here 1. true variance between the lectures, 2. systematic error variance in the assessment of the lecture by the two raters, and 3. the unsystematic error variance) and their relative proportion (Clausen et al., 2013; Hugener et al., 2006). A high percentage of the variance component *video* and a low percentage of the variance component *rater* is considered desirable by Lotz et al. (2013) as this suggests that the judgments are due to differences in lectures rather than raters. The majority of the total variance in the rating judgments could be explained by true differences between lectures (61.8 – 69.5%), while systematic variance between raters explains only a proportion up to 0.5%. Another large explanatory proportion of the total variance was attributable to unsystematic variance (30.5 – 38.2%), which reflects the component of interaction between the lecture and the rater. The relative generalizability coefficient reports the ratio of true score variance to observed score variance, analogous to classical test theory (Renkl et al., 1993) and is applied

when the agreement in rank order rather than the absolute level of the judgments is crucial (Lotz et al., 2013). It can be interpreted equivalently to the reliability coefficient from the classical test theory (Wirtz et al., 2002). We followed Lotz et al. (2013), who established a relative generalizability coefficient of > .70 as a criterion for sufficient data quality[2]. In this study, the relative generalizability coefficient for all three indicators ranged from .76 to .82.

Since it is discussable whether the three-point scale for assessing the clarity of instruction is truly metric, we additionally computed Cicchetti-Allison weighted kappas because they are applicable to ordinal data coded by two raters (Gisev et al., 2013). In this extension of Cohen's kappa, weights are assigned to categories such that similar categories receive a higher agreement than less similar categories (Cohen, 1968). In line with the interpretation of Landis et al. (1977), kappa values of .41-.60 indicate moderate agreement, .61-.80 substantial agreement and .81-1.00 almost perfect agreement. In this study, the confidence intervals for Cohen's kappa coefficients (κ) for all three indicators yielded values ranged from .45 to .73, indicating moderate to substantial interrater agreement.

[2] Other studies report a minimum value of >.65 (Hugener et al., 2006).

Table B.4 *Intra-class correlation, variance components and relative generalizability coefficients, weighted kappa for the 3 rating dimensions*

Item	*n*	*ICC*	*95% CI*	$g_{relative}$	variance components			weighted κ	95% CI
					video	rater	v*r + ε		
Detail	238	.69	.62 – .75	.82	69.5%	0.0%	30.5%	.65	.56 – .73
Variation	238	.63	.55 – .70	.78	63.3%	0.5%	36.2%	.59	.50 – .68
Logical Inconsistency	238	.62	.53 – .69	.76	61.8%	0.0%	38.2%	.59	.45 – .73

Note. n = video observations, ICC = intra-class correlation, $g_{relative}$= relative generalizability coefficient, weighted κ = weighted kappa, v*r + ε = variance component of video and rater interaction plus unsystematic variation, 95% CI = 95% confidence interval.

B.5 Two-level model

Due to the worse model fit of the complex model (which includes all indicators of instructional clarity), we decided to report the simpler models, i.e., each model includes only one indicator of instructional clarity. The *ppp*-values ranged between .390 and .402 and the 95% CI for the difference between observed and replicated chi-squared values comprises zero, indicating an adequate model fit (Neelon et al., 2010).

Table B.5 *Two-level models for decomposing the variance of instructional clarity within situations and between time points*

	**Model 1**<br>Detail of Explanation	**Model 2**<br>Variation of Explanation	**Model 3**<br>Logical Inconsistency
	Estimates [*CI*]	Estimates [*CI*]	Estimates [*CI*]
**Fixed effects**			
Intercept	1.093 [0.835; 1.350]	0.728 [0.570; 0.883]	0.177 [0.045; 0.308]
**Random parameters**			
σ^2 (situation-level)	.421 [.352; .511]	.378 [.316; .459]	.135 [.113; .164]
σ^2 (time point-level)	.096 [.024; .487]	.026 [.003; .175]	.024 [.005; .119]
ICC			
time point-level	.186 [.053; .537]	.065 [.007; .317]	.150 [.036; .472]
ppp-value	.402 [-7.879; 11.957]	.390 [-7.556; 12.300]	.399 [-7.975; 12.150]

Note. For the fixed effect estimates, cell entries are parameter (beta) estimates and CI = 95% credibility intervals. Random effects are presented as estimates and credibility intervals. The *ppp*-value refers to posterior predictive p-value, a measure of model fit.

References

Clausen, M., Reusser, K. & Klieme, E. (2003). Unterrichtsqualität auf der Basis hoch inferenter Unterrichtsbeurteilungen. Ein Vergleich zwischen Deutschland und der deutschsprachigen Schweiz. *Unterrichtswissenschaft*, *31*(2), 122-141. https://doi.org/10.25656/01:6775

Cohen, J. (1968). Weighted kappa: nominal scale agreement provision for scaled disagreement or partial credit. *Psychological bulletin*, *70*(4), 213. https://doi.org/10.1037/h0026256

Gisev, N., Bell, J. S., & Chen, T. F. (2013). Interrater agreement and interrater reliability: key concepts, approaches, and applications. *Research in Social and Administrative Pharmacy*, *9*(3), 330-338. https://doi.org/10.1016/j.sapharm.2012.04.004

Hugener, I., Pauli, C., & Reusser, K. (2006). *Dokumentation der Erhebungs-und Auswertungsinstrumente zur schweizerisch-deutschen Videostudie „Unterrichtsqualität, Lernverhalten und mathematisches Verständnis". 3.Videoanalysen*. GFPF/DIPF. https://doi.org/10.25656/01:3130

Koo, T. K., & Li, M. Y. (2016). A Guideline of Selecting and Reporting Intraclass Correlation Coefficients for Reliability Research. *Journal of chiropractic medicine*, *15*(2), 155–163. https://doi.org/10.1016/j.jcm.2016.02.012

Landis, J. R., & Koch, G. G. (1977). The measurement of observer agreement for categorical data. *Biometrics*, *33*(1), 159-174. https://doi.org/10.2307/2529310Liborius, P., Bellhäuser, H., & Schmitz, B. (2019). What makes a good study day? An intraindividual study on university students' time investment by means of time-series analyses. *Learning and Instruction*, *60*, 310-321. https://doi.org/10.1016/j.learninstruc.2017.10.006

Lotz, M., Lipowsky, F., & Faust, G. (2013). *Dokumentation der Erhebungsinstrumente des Projekts „Persönlichkeits-und Lernentwicklung von Grundschulkindern" (PERLE). 3. Technischer Bericht zu den PERLE-Videostudien*. GFPF. https://doi.org/10.25656/01:7702

Neelon, B. H., O'Malley, A. J., & Normand, S. L. T. (2010). A Bayesian model for repeated measures zero-inflated count data with application to outpatient psychiatric service use. *Statistical modelling*, *10*(4), 421-439. 10.1177/1471082X0901000404

Renkl, A. & Helmke, A. (1993). Prinzip, Nutzen und Grenzen der Generalisierungstheorie. *Empirische Pädagogik*, 7(1), 63–85.

Wirtz, M. & Caspar, F. (2002). *Beurteilerübereinstimmung und Beurteilerreliabilität: Methoden zur Bestimmung und Verbesserung der Zuverlässigkeit von Einschätzungen mittels*
