# Supplementary material for: Student motivation and instructional clarity: Linking experience sampling method data to objective behavioural observations
Source: Br J Educ Psychol. 2025 Apr 18;95(Suppl 1):S281–99. doi: 10.1111/bjep.12775 (PMC12427158; doi:10.1111/bjep.12775)
Supplement: Supplementary file 2 — Appendix S2: [file BJEP-95-S281-s002.zip › Appendix C - Cross-classified multilevel models/Appendix C - Cross-classified multilevel models.pdf]

Appendix C – Cross-classified Multilevel Models

Bayesian estimation techniques were chosen, because it can be even applied in small samples, provides proper parameter estimates by preventing implausible values, and always converges whenever enough iterations are considered (Hox et al., 2018; Van de Schoot et al., 2014). Based on the Markov chain Monte Carlo (MCMC) algorithm, the variances can be estimated by an iterative approximation to the posterior distribution of the parameters (Muthén et al., 2012). For each parameter, point estimates and a 95%-credibility interval were calculated to assess statistical significance. The following settings were applied: We set a random seed value of 12,345 for analyses, used four MCMC-chains and a thinning of 10 (i.e., every 10th iteration was recorded) and relied on the default settings of the prior distribution. The default for regression parameters were normal distributions with a prior mean of zero and an infinitive large prior variance; the default for (residual) variance parameters were non-informative inverse gamma distribution with an infinity prior mean and an infinity large prior variance resulting in a positive distribution (Asparouhov et al., 2010; Van de Schoot et al., 2014). A minimum of 10,000 iterations (and 5,000 iterations for null-CCMM) were performed to fulfill the convergence criterion, taking the first half of the iterations as a burn-in phase before reaching the target distribution. By default, M*plus* used Gelman-Rubin potential scale reduction (Gelman et al., 1992) to assess the convergence of the chain (converged when PSR value = .05). We changed the default criterion to a stricter value (PSR value = .001). To inspect the convergence of all chains in a visual way, we examined trace plots, including the convergence of chains to the same target distribution and stability of chains across all iterations (Van de Schoot et al., 2014). In order to evaluate the model fit, posterior predictive checking can be carried out (Gelman et al., 2004). This tests the deviation between the data simulated by the model and the actual observed data, which should be as small as possible (Van de Schoot et al., 2014). A good model fit is reflected in a Bayesian posterior predictive p-value (ppp-value), calculated via χ^2-difference values, of 0.5 and a confidence interval that includes zero. In addition, we checked Bayesian autocorrelations plots and Bayesian posterior parameter distribution.

The data were prepared in R (R Development Core Team, 2008), statistical analyses were performed using M*plus* version 8.8 software program (Muthén et al., 1998 – 2017).

This Appendix contains the results of supplementary CCMMs for each SEVT facets (expectation of success, competence beliefs, intrinsic value, attainment value, utility value, effort costs, emotional costs and opportunity costs) as well as for each observation interval (first, second and third interval prior to a beep[1]).

Overview

Mean observation interval[2]	
Table C.1.1	Shows the results of the CCMMs for each SEVT facet[3]
Table C.1.2	Shows zero-order correlations among each SEVT facet and instructional clarity at learning situation-level
First observation interval (6 – 9 minutes before the beep)	
Table C.2.1	Shows the results of the CCMMs for each SEVT composite components[4]
Table C.2.2	Shows zero-order correlations among each SEVT composite component and instructional clarity at learning situation-level
Table C.2.3	Shows the results of the CCMMs for each SEVT facet
Table C.2.4	Shows zero-order correlations among each SEVT facet and instructional clarity at learning situation-level
Second observation interval (3 – 6 minutes before the beep)	
Table C.3.1	Shows the results of the CCMMs for each SEVT composite components
Table C.3.2	Shows zero-order correlations among each SEVT composite component and instructional clarity at learning situation-level
Table C.3.3	Shows the results of the CCMMs for each SEVT facet
Table C.3.4	Shows zero-order correlations among each SEVT facet and instructional clarity at learning situation-level
Third observation interval (0 – 3 minutes before the beep)	
Table C.4.1	Shows the results of the CCMMs for each SEVT composite components
Table C.4.2	Shows zero-order correlations among each SEVT composite component and instructional clarity at learning situation-level
Table C.4.3	Shows the results of the CCMMs for each SEVT facet
Table C.4.4	Shows zero-order correlations among each SEVT facet and instructional clarity at learning situation-level

[1]The participants were beeped every nine minutes to complete the motivational questionnaire. In parallel, a time sampling schedule was used to rate the videos for instructional clarity at three-minute intervals. The start of a coding interval refers to the associated signal, i.e., three, six or nine minutes before the beep. The first interval starts nine minutes before the beep, the second interval starts six minutes before the beep and the third interval starts three minutes before the beep.

[2] Instructional clarity was *averaged* across three observation intervals prior to a beep.

[3] SEVT facets: expectation of success, competence beliefs, intrinsic value, attainment value, utility value, effort costs, emotional costs and opportunity costs.

[4] SEVT component: situational expectancies, values and costs.

C.1 Results of the mean observation interval

Eight supplementary CCMMs of the individual SEVT facets were conducted, see Table C.1.1 for variance component estimates and Table C.1.2 for zero-order correlations at learning situation-level. The three indicators of instructional clarity were averaged across three observation intervals prior to a beep and included as learning situation-level covariates. All models fitted the data very well, i.e., the *ppp*-value is close to .5 and the 95% CI for the difference between the observed and the replicated χ^2 included zero.

In line with our hypotheses, situations with detailed explanations positively related to expectation of success (r = .448). No other correlations were found. For all models, excluding opportunity cost, most of the variance was located at the within-level (52.7 – 69.1%), followed by between-student level (25.9 – 43.8%). A relatively large amount of variance in opportunity costs was attributable to variation in student-level (59.5%) and less in fluctuations within students and learning situations (39.1%). When between-level covariate instructional clarity was added to the models, the within-level variance remained consistent compared to null model (Table A.4.1). The between student-level variance declined by up to .055 and the between learning situation-level variance declined by up to .010 relative to null model. Compared to the models with composite components (see Table 2 in the publication), the models with individual SEVT facets explained more variance at the within-level (up to .063 more explained variance in expectations, .127 in values and .200 in costs) and at the between-student level (up to .019 more explained variance in expectations, .058 in values and .174 in costs). The explained variance at between-learning situations level did not substantially differ from the models with composite components (up to .007 more explained variance in expectations, .009 in values and .012 in costs).

Table C.1.1 *Cross-classified multilevel analyses results for predicting students' situational motivation from instructional clarity*

	**M1.a** Expectation of success<br>Estimates [*CI*]	**M1.b** Competence beliefs<br>Estimates [*CI*]	
**Fixed effects**			
Intercept	2.901 [2.799; 3.005]	3.295 [3.163; 3.425]	
Learning situation-level			
Detail	.080 [.020; .140]	-.014 [-.093; .064]	
Variation	.005 [-.058; .068]	-.034 [-.117; .050]	
Logical Inconsistency	-.009 [-.124; .106]	-.113 [-.261; .037]	
**Random parameters**			
σ^2 (response-level)	.220 [.206; .235]	.229 [.215; .245]	
σ^2 (student-level)	.099 [.074; .133]	.103 [.077; .140]	
σ^2 (learning situation-level)	.006 [.002; .012]	.016 [.009; .026]	
ICC			
student-level	.306 [.245; .374]	.297 [.236; .365]	
learning situation-level	.017 [.005; .037]	.045 [.026; .074]	
ppp-value	.482 [-11.722; 11.972]	.496 [-12.049; 12.953]	
	**M2.a** Intrinsic value<br>Estimates [*CI*]	**M2.b** Attainment value<br>Estimates [*CI*]	**M2.c** Utility value<br>Estimates [*CI*]
**Fixed effects**			
Intercept	2.944 [2.793; 3.095]	2.881 [2.755; 3.011]	3.106 [2.971; 3.240]
Learning situation-level			
Detail	.024 [-.070; .116]	.018 [-.055; .089]	-.003 [-.082; .075]
Variation	-.030 [-.128; .067]	.029 [-.048; .105]	.051 [-.033; .133]
Logical Inconsistency	-.016 [-.190; .157]	-.035 [-.173; .103]	-.055 [-.204; .094]
**Random parameters**			
σ^2 (response-level)	.304 [.285; .325]	.273 [.256; .292]	.268 [.251; .286]
σ^2 (student-level)	.114 [.084; .155]	.183 [.141; .241]	.155 [.118; .207]
σ^2 (learning situation-level)	.022 [.013; .037]	.010 [.004; .019]	.014 [.007; .025]
ICC			
student-level	.259 [.203; .323]	.393 [.328; .464]	.355 [.292; .425]
learning situation-level	.050 [.029; .083]	.021 [.008; .041]	.032 [.017; .056]
ppp-value	.483 [-11.457; 11.987]	.492 [-11.417; 12.284]	.487 [-12.673; 12.787]
	**M3.a** Effort costs<br>Estimates [*CI*]	**M3.b** Emotional costs<br>Estimates [*CI*]	**M3.c** Opportunity costs<br>Estimates [*CI*]
**Fixed effects**			
Intercept	1.955 [1.783; 2.129]	1.618 [1.480; 1.759]	1.770 [1.624; 1.923]
Learning situation-level			
Detail	-.068 [-.167; .030]	.002 [-.074; .080]	-.023 [-.094; .050]
Variation	.091 [-.012; .196]	.013 [-.070; .095]	.021 [-.054; .097]
Logical Inconsistency	.034 [-.154; .219]	-.071 [-.224; .078]	.058 [-.079; .193]
**Random parameters**			
σ^2 (response-level)	.366 [.343; .391]	.283 [.265; .302]	.266 [.249; .284]
σ^2 (student-level)	.304 [.235; .398]	.197 [.151; .261]	.406 [.319; .527]
σ^2 (learning situation-level)	.024 [.014; .040]	.013 [.006; .024]	.010 [.004; .019]
ICC			
student-level	.438 [.373; .507]	.400 [.335; .470]	.595 [.533; .658]
learning situation-level	.035 [.020; .057]	.026 [.013; .048]	.014 [.006; .028]
ppp-value	.490 [-12.135; 13.129]	.485 [-12.067; 12.537]	.489 [-13.164; 12.242]

Note. For the fixed effect estimates, cell entries are parameter (beta) estimates and CI = 95% credibility intervals. Random effects are presented as estimates and credibility intervals. The *ppp*-value refers to posterior predictive p-value, a measure of model fit.

Table C.1.2 *Zero-order correlations among students' situational motivation and instructional clarity at learning situation-level 2b*

Model		Detail	Variation	Logical Inconsistency
		Estimates [*CI*]	Estimates [*CI*]	Estimates [*CI*]
M1.a	Expectation of success	.448 [.117; .739]	.021 [-.306; .351]	-.033 [-.384; .303]
M1.b	Competence beliefs	-.053 [-.321; .224]	-.118 [-.383; .168]	-.227 [-.487; .074]
M2.a	Intrinsic value	.075 [-.203; .351]	-.093 [-.372; .192]	-.033 [-.326; .257]
M2.b	Attainment value	.085 [-.241; .413]	.125 [-.208; .444]	-.087 [-.432; .248]
M2.c	Utility value	-.014 [-.309; .287]	.183 [-.118; .462]	-.121 [-.421; .188]
M3.a	Effort costs	-.197 [-.446; .082]	.244 [-.035; .490]	.053 [-.238; .337]
M3.b	Emotional costs	.017 [-.288; .324]	.053 [-.252; .358]	-.156 [-.451; .176]
M3.c	Opportunity costs	-.110 [-.433; .215]	.091 [-.239; .405]	.147 [-.201; .482]

Note. CI = Credibility Interval.

C.2 Results of the first observation interval (6 – 9 minutes before the beep)

Supplementary CCMMs of the SEVT components were conducted (see Table C.2.1 for variance component estimates and Table C.2.2 for zero-order correlations at learning situation-level) as well as eight supplementary CCMMs of the individual SEVT facets (see Table C.2.3 for variance component estimates and Table C.2.4 for zero-order correlations at learning situation-level). Instructional clarity of the first observation interval was included as learning situation-level covariates. The *ppp*-values ranged from .478 to .503 and the 95% CIs for the difference between observed and replicated chi-squared values comprises zero, indicating excellent model fits.

As anticipated, detail of explanation predicted variability in perceived costs (β = -.047), especially effort costs (β = -.087) as well as expectation of success (β = .068) in the first observation interval. As opposed to our expectation, variation in explanation and logical inconsistency could not explain any part of the variance.

Table C.2.1 *Cross-classified multilevel analyses results for predicting students' situational motivation from instructional clarity*

	**M1_1** Expectancies	**M2_1** Values	**M3_1** Costs
	Estimates [*CI*]	Estimates [*CI*]	Estimates [*CI*]
**Fixed effects**			
Intercept	3.084 [3.006; 3.162]	2.964 [2.87; 3.056]	1.838 [1.734; 1.944]
Learning situation-level			
Detail	.040 [-.002; .081]	.027 [-.022; .074]	-.047 [-.094; -.001]
Variation	-.017 [-.064; .031]	.010 [-.046; .065]	.007 [-.045; .061]
Logical Inconsistency	-.022 [-.090; .049]	-.023 [-.101; .059]	-.016 [-.093; .061]
**Random parameters**			
σ^2 (response-level)	.166 [.155; .177]	.177 [.166; .189]	.167 [.156; .179]
σ^2 (student-level)	.082 [.061; .111]	.125 [.095; .166]	.23 [.180; .300]
σ^2 (learning situation-level)	.009 [.004; .015]	.014 [.008; .023]	.012 [.007; .020]
ICC			
student-level	.319 [.257; .390]	.396 [.330; .467]	.562 [.498; .628]
learning situation-level	.033 [.017; .059]	.043 [.024; .071]	.029 [.017; .049]
ppp-value	.485 [-12.123; 11.841]	.484 [-12.083; 11.657]	.487 [-12.092; 11.824]

Note. For the fixed effect estimates, cell entries are parameter (beta) estimates and CI = 95% credibility intervals. Random effects are presented as estimates and credibility intervals. The *ppp*-value refers to posterior predictive p-value, a measure of model fit.

Table C.2.2 *Zero-order correlations among students' situational motivation and instructional clarity at learning situation-level 2b*

Model		Detail	Variation	Logical Inconsistency
		Estimates [*CI*]	Estimates [*CI*]	Estimates [*CI*]
M1_1	Expectancies	.293 [-.010; .552]	-.111 [-.386; .187]	-.103 [-.396; .208]
M2_1	Values	.162 [-.127; .426]	.050 [-.232; .334]	-.091 [-.377; .200]
M3_1	Costs	-.2901 [-.545; -.003]	.045 [-.242; .314]	-.056 [-.338; .233]

Note. CI = Credibility Interval.

Table C.2.3 *Cross-classified multilevel analyses results for predicting students' situational motivation from instructional clarity*

	**M1.a_1** Expectation of success<br>Estimates [*CI*]	**M1.b_1** Competence beliefs<br>Estimates [*CI*]	
**Fixed effects**			
Intercept	2.944 [2.866; 3.023]	3.229 [3.131; 3.325]	
Learning situation-level			
Detail	.068 [.029; .108]	.009 [-.046; .062]	
Variation	-.033 [-.079; .012]	.002 [-.059; .065]	
Logical Inconsistency	-.031 [-.096; .038]	-.011 [-.100; .080]	
**Random parameters**			
σ^2 (response-level)	.221 [.207; .237]	.229 [.214; .245]	
σ^2 (student-level)	.098 [.073; .133]	.102 [.076; .139]	
σ^2 (learning situation-level)	.005 [.001; .011]	.017 [.010; .028]	
ICC			
student-level	.302 [.242; .372]	.292 [.232; .363]	
learning situation-level	.015 [.003; .034]	.049 [.028; .080]	
ppp-value	.498 [-12.281; 12.050]	.486 [-12.267; 12.546]	

	**M2.a_1** Intrinsic value<br>Estimates [*CI*]	**M2.b_1** Attainment value<br>Estimates [*CI*]	**M2.c_1** Utility value<br>Estimates [*CI*]
**Fixed effects**			
Intercept	2.915 [2.804; 3.024]	2.885 [2.782; 2.987]	3.096 [2.993; 3.199]
Learning situation-level			
Detail	.037 [-.026; .099]	.028 [-.022; .077]	.013 [-.041; .065]
Variation	-.010 [-.082; .062]	.004 [-.052; .061]	.038 [-.023; .099]
Logical Inconsistency	-.017 [-.118; .088]	-.019 [-.101; .067]	-.033 [-.121; .058]
**Random parameters**			
σ^2 (response-level)	.305 [.285; .326]	.273 [.255; .292]	.267 [.250; .286]
σ^2 (student-level)	.114 [.084; .155]	.183 [.140; .242]	.154 [.116; .207]
σ^2 (learning situation-level)	.022 [.012; .038]	.010 [.004; .020]	.014 [.007; .025]
ICC			
student-level	.258 [.202; .324]	.393 [.328; .463]	.353 [.289; .425]
learning situation-level	.051 [.029; .084]	.022 [.009; .043]	.032 [.016; .056]
ppp-value	.503 [-12.059; 12.016]	.478 [-12.127; 12.112]	.487 [-12.507; 12.219]

	**M3.a_1** Effort costs<br>Estimates [*CI*]	**M3.b_1** Emotional costs<br>Estimates [*CI*]	**M3.c_1** Opportunity costs<br>Estimates [*CI*]
**Fixed effects**			
Intercept	2.056 [1.923; 2.19]	1.64 [1.532; 1.749]	1.798 [1.674; 1.925]
Learning situation-level			
Detail	-.087 [-.154; -.023]	-.018 [-.071; .035]	-.042 [-.089; .005]
Variation	.001 [-.072; .076]	.005 [-.058; .067]	.009 [-.044; .063]
Logical Inconsistency	-.050 [-.159; .060]	-.038 [-.126; .052]	.036 [-.043; .117]
**Random parameters**			
σ^2 (response-level)	.368 [.344; .393]	.283 [.265; .302]	.268 [.251; .287]
σ^2 (student-level)	.302 [.232; .399]	.195 [.149; .260]	.400 [.313; .521]
σ^2 (learning situation-level)	.023 [.013; .038]	.014 [.007; .025]	.008 [.003; .017]
ICC			
student-level	.436 [.369; .507]	.397 [.331; .468]	.590 [.527; .655]
learning situation-level	.033 [.018; .054]	.028 [.014; .050]	.012 [.004; .026]
ppp-value	.488 [-12.729; 12.340]	.495 [-12.408; 13.077]	.487 [-11.936; 12.183]

Note. For the fixed effect estimates, cell entries are parameter (beta) estimates and CI = 95% credibility intervals. Random effects are presented as estimates and credibility intervals. The *ppp*-value refers to posterior predictive p-value, a measure of model fit.

Table C.2.4 *Zero-order correlations among students' situational motivation and instructional clarity at learning situation-level 2b*

Model		Detail	Variation	Logical Inconsistency
		Estimates [*CI*]	Estimates [*CI*]	Estimates [*CI*]
M1.a_1	Expectation of success	.553 [.241; .813]	-.232 [-.509; .094]	-.152 [-.463; .190]
M1.b_1	Competence beliefs	.052 [-.232; .335]	.008 [-.284; .301]	-.044 [-.341; .254]
M2.a_1	Intrinsic value	.178 [-.105; .444]	-.045 [-.317; .242]	-.061 [-.346; .238]
M2.b_1	Attainment value	.191 [-.146; .511]	.033 [-.294; .364]	-.081 [-.425; .266]
M2.c_1	Utility value	.074 [-.226; .369]	.200 [-.112; .485]	-.119 [-.421; .192]
M3.a_1	Effort costs	-.381 [-.607; -.098]	.007 [-.276; .283]	-.132 [-.406; .152]
M3.b_1	Emotional costs	-.112 [-.410; .203]	.024 [-.292; .333]	-.146 [-.443; .185]
M3.c_1	Opportunity costs	-.302 [-.607; .042]	.065 [-.276; .381]	.177 [-.180; .498]

Note. CI = Credibility Interval.

C.3 Results of the second observation interval (3 – 6 minutes before the beep)

Supplementary CCMMs of the SEVT components were conducted (see Table C.3.1 for variance component estimates and Table C.3.2 for zero-order correlations at learning situation-level) as well as eight supplementary CCMMs of the individual SEVT facets (see Table C.3.3 for variance component estimates and Table C.3.4 for zero-order correlations at learning situation-level). Instructional clarity of the second observation interval was included as learning situation-level covariates. The *ppp*-values ranged from .460 to .479 and the 95% CIs for the difference between observed and replicated chi-squared values comprises zero, indicating excellent model fits.

All three indicators of instructional clarity (detail of explanation, variation of explanation and logical inconsistency) were not associated with expectancies, nor with task values and costs at learning situation-level. The same applied to the facets of SEVT.

Table C.3.1 *Cross-classified multilevel analyses results for predicting students' situational motivation from instructional clarity*

	**M1_2** Expectancies	**M2_2** Values	**M3_2** Costs
	Estimates [*CI*]	Estimates [*CI*]	Estimates [*CI*]
**Fixed effects**			
Intercept	3.130 [3.046; 3.215]	3.005 [2.908; 3.104]	1.744 [1.636; 1.856]
Learning situation-level			
Detail	-.013 [-.059; .033]	-.034 [-.087; .017]	.030 [-.020; .080]
Variation	.003 [-.046; .051]	.031 [-.026; .085]	.019 [-.035; .072]
Logical Inconsistency	-.049 [-.120; .022]	.007 [-.073; .087]	.015 [-.063; .092]
**Random parameters**			
σ^2 (response-level)	.165 [.155; .177]	.177 [.166; .189]	.167 [.156; .178]
σ^2 (student-level)	.084 [.063; .112]	.125 [.096; .165]	.233 [.182; .303]
σ^2 (learning situation-level)	.009 [.005; .016]	.013 [.008; .022]	.012 [.007; .020]
ICC			
student-level	.324 [.261; .392]	.396 [.331; .467]	.565 [.502; .631]
learning situation-level	.035 [.018; .061]	.041 [.024; .069]	.03 [.017; .049]
ppp-value	.469 [-12.342; 12.643]	.474 [-12.299; 12.677]	.473 [-12.382; 12.576]

Note. Instructional clarity of the second interval was included in the analyses. For the fixed effect estimates, cell entries are parameter (beta) estimates and CI = 95% credibility intervals. Random effects are presented as estimates and credibility intervals. The *ppp*-value refers to posterior predictive p-value, a measure of model fit.

Table C.3.2 *Zero-order correlations among students' situational motivation and instructional clarity at learning situation-level 2b*

Model		Detail	Variation	Logical Inconsistency
		Estimates [*CI*]	Estimates [*CI*]	Estimates [*CI*]
M1_2	Expectancies	-.077 [-.363; .220]	.020 [-.265; .300]	-.208 [-.478; .095]
M2_2	Values	-.183 [-.441; .099]	.157 [-.118; .414]	.028 [-.241; .301]
M3_2	Costs	.176 [-.110; .437]	.107 [-.165; .370]	.056 [-.223; .326]

Note. CI = Credibility Interval.

Table C.3.3 *Cross-classified multilevel analyses results for predicting students' situational motivation from instructional clarity*

	**M1.a_2** Expectation of success<br>Estimates [*CI*]	**M1.b_2** Competence beliefs<br>Estimates [*CI*]	
**Fixed effects**			
Intercept	2.977 [2.89; 3.065]	3.287 [3.183; 3.391]	
Learning situation-level			
Detail	.005 [-.042; .051]	-.030 [-.089; .028]	
Variation	.014 [-.036; .063]	-.009 [-.072; .053]	
Logical Inconsistency	-.024 [-.096; .048]	-.067 [-.158; .023]	
**Random parameters**			
σ^2 (response-level)	.219 [.206; .234]	.229 [.215; .245]	
σ^2 (student-level)	.100 [.074; .135]	.103 [.077; .139]	
σ^2 (learning situation-level)	.007 [.003; .015]	.016 [.009; .027]	
ICC			
student-level	.306 [.245; .375]	.295 [.236; .364]	
learning situation-level	.022 [.008; .044]	.046 [.027; .076]	
ppp-value	.473 [-11.991; 13.812]	.460 [-12.120; 13.209]	
	**M2.a_2** Intrinsic value<br>Estimates [*CI*]	**M2.b_2** Attainment value<br>Estimates [*CI*]	**M2.c_2** Utility value<br>Estimates [*CI*]
**Fixed effects**			
Intercept	2.968 [2.85; 3.087]	2.918 [2.811; 3.026]	3.128 [3.021; 3.238]
Learning situation-level			
Detail	-.034 [-.104; .032]	-.029 [-.083; .023]	-.035 [-.093; .022]
Variation	.009 [-.065; .080]	.033 [-.024; .089]	.050 [-.013; .111]
Logical Inconsistency	.023 [-.081; .129]	.005 [-.076; .088]	-.005 [-.094; .084]
**Random parameters**			
σ^2 (response-level)	.304 [.285; .325]	.272 [.255; .290]	.269 [.252; .287]
σ^2 (student-level)	.114 [.084; .154]	.183 [.140; .240]	.155 [.117; .206]
σ^2 (learning situation-level)	.023 [.013; .038]	.01 [.004; .020]	.014 [.007; .025]
ICC			
student-level	.258 [.203; .323]	.393 [.328; .463]	.353 [.290; .424]
learning situation-level	.051 [.030; .084]	.022 [.009; .042]	.031 [.016; .055]
ppp-value	.479 [-11.752; 13.470]	.469 [-11.512; 13.236]	.463 [-11.905; 13.302]
	**M3.a_2** Effort costs<br>Estimates [*CI*]	**M3.b_2** Emotional costs<br>Estimates [*CI*]	**M3.c_2** Opportunity costs<br>Estimates [*CI*]
**Fixed effects**			
Intercept	1.873 [1.733; 2.015]	1.574 [1.463; 1.688]	1.761 [1.631; 1.894]
Learning situation-level			
Detail	.023 [-.048; .093]	.042 [-.014; .100]	.020 [-.032; .073]
Variation	.068 [-.007; .143]	.004 [-.056; .064]	-.017 [-.073; .039]
Logical Inconsistency	.052 [-.057; .163]	-.014 [-.102; .072]	.016 [-.066; .098]
**Random parameters**			
σ^2 (response-level)	.366 [.343; .391]	.283 [.265; .303]	.266 [.249; .285]
σ^2 (student-level)	.306 [.236; .402]	.197 [.151; .260]	.406 [.319; .527]
σ^2 (learning situation-level)	.023 [.013; .039]	.013 [.006; .023]	.010 [.004; .020]
ICC			
student-level	.439 [.374; .510]	.399 [.335; .470]	.595 [.533; .658]
learning situation-level	.033 [.019; .055]	.026 [.012; .047]	.015 [.006; .029]
ppp-value	.469 [-12.088; 13.071]	.474 [-11.967; 12.960]	.473 [-12.141; 13.358]

Note. For the fixed effect estimates, cell entries are parameter (beta) estimates and CI = 95% credibility intervals. Random effects are presented as estimates and credibility intervals. The *ppp*-value refers to posterior predictive p-value, a measure of model fit.

Table C.3.4 *Zero-order correlations among students' situational motivation and instructional clarity at learning situation-level 2b*

Model		Detail	Variation	Logical Inconsistency
		Estimates [*CI*]	Estimates [*CI*]	Estimates [*CI*]
M1.a_2	Expectation of success	.045 [-.294; .377]	.106 [-.227; .417]	-.112 [-.444; .223]
M1.b_2	Competence beliefs	-.142 [-.400; .147]	-.039 [-.305; .235]	-.210 [-.464; .068]
M2.a_2	Intrinsic value	-.136 [-.400; .138]	.040 [-.230; .308]	.069 [-.205; .336]
M2.b_2	Attainment value	-.176 [-.474; .147]	.192 [-.122; .481]	.023 [-.287; .335]
M2.c_2	Utility value	-.18 [-.454; .112]	.246 [-.043; .507]	-.017 [-.302; .268]
M3.a_2	Effort costs	.093 [-.188; .360]	.259 [-.011; .508]	.139 [-.147; .406]
M3.b_2	Emotional costs	.233 [-.074; .507]	.024 [-.269; .322]	-.050 [-.351; .251]
M3.c_2	Opportunity costs	.134 [-.188; .438]	-.106 [-.412; .219]	.067 [-.251; .381]

Note. CI = Credibility Interval.

C.4 Results of the third observation interval (0 – 3 minutes before the beep)

Supplementary CCMMs of the SEVT components were conducted (see Table C.4.1 for variance component estimates and Table C.4.2 for zero-order correlations at learning situation-level) as well as eight supplementary CCMMs of the individual SEVT facets (see Table C.4.3 for variance component estimates and Table C.4.4 for zero-order correlations at learning situation-level). Instructional clarity of the second observation interval was included as learning situation-level covariates. The *ppp*-values ranged from .469 to .485 and the 95% CIs for the difference between observed and replicated chi-squared values comprises zero, indicating excellent model fits.

As anticipated, detail of explanation predicted variability in expectation of success (β = .041) in the third observation interval. As opposed to our expectation, variation in explanation and logical inconsistency could not explain any part of the variance.

Table C.4.1 *Cross-classified multilevel analyses results for predicting students' situational motivation from instructional clarity*

	**M1_3** Expectancies	**M2_3** Values	**M3_3** Costs
	Estimates [*CI*]	Estimates [*CI*]	Estimates [*CI*]
**Fixed effects**			
Intercept	3.097 [3.017; 3.177]	2.998 [2.905; 3.091]	1.779 [1.674; 1.886]
Learning situation-level			
Detail	.020 [-.021; .062]	.017 [-.030; .063]	-.014 [-.059; .031]
Variation	-.012 [-.058; .035]	-.023 [-.075; .030]	.038 [-.014; .089]
Logical Inconsistency	-.017 [-.115; .083]	-.059 [-.170; .052]	-.002 [-.110; .106]
**Random parameters**			
σ^2 (response-level)	.166 [.155; .177]	.177 [.166; .189]	.166 [.156; .178]
σ^2 (student-level)	.084 [.063; .112]	.125 [.096; .166]	.234 [.182; .304]
σ^2 (learning situation-level)	.009 [.005; .016]	.013 [.007; .022]	.012 [.007; .020]
ICC			
student-level	.323 [.261; .392]	.397 [.332; .468]	.566 [.502; .632]
learning situation-level	.036 [.019; .062]	.041 [.024; .069]	.030 [.017; .050]
ppp-value	.471 [-11.425; 12.605]	.472 [-11.434; 12.572]	.472 [-11.439; 12.779]

Note. For the fixed effect estimates, cell entries are parameter (beta) estimates and CI = 95% credibility intervals. Random effects are presented as estimates and credibility intervals. The *ppp*-value refers to posterior predictive p-value, a measure of model fit.

Table C.4.2 *Zero-order correlations among students' situational motivation and instructional clarity at learning situation-level 2b*

Model		Detail	Variation	Logical Inconsistency
		Estimates [*CI*]	Estimates [*CI*]	Estimates [*CI*]
M1_3	Expectancies	.157 [-.155; .433]	-.076 [-.367; .224]	-.047 [-.355; .262]
M2_3	Values	.111 [-.173; .378]	-.121 [-.379; .161]	-.155 [-.428; .140]
M3_3	Costs	-.084 [-.353; .196]	.224 [-.065; .475]	-.001 [-.286; .286]

Note. CI = Credibility Interval.

Table C.4.3 *Cross-classified multilevel analyses results for predicting students' situational motivation from instructional clarity*

	**M1.a_3** Expectation of success<br>Estimates [*CI*]	**M1.b_3** Competence beliefs<br>Estimates [*CI*]	
**Fixed effects**			
Intercept	2.932 [2.851; 3.014]	3.265 [3.168; 3.363]	
Learning situation-level			
Detail	.041 [.000; .081]	.003 [-.049; .056]	
Variation	.009 [-.036; .055]	-.035 [-.094; .023]	
Logical Inconsistency	.031 [-.066; .129]	-.057 [-.180; .068]	
**Random parameters**			
σ^2 (response-level)	.220 [.207; .235]	.229 [.215; .245]	
σ^2 (student-level)	.100 [.075; .134]	.103 [.077; .139]	
σ^2 (learning situation-level)	.006 [.002; .013]	.016 [.009; .027]	
ICC			
student-level	.305 [.245; .373]	.295 [.236; .365]	
learning situation-level	.020 [.006; .040]	.047 [.027; .077]	
ppp-value	.477 [-11.816; 12.701]	.476 [-11.552; 13.719]	
	**M2.a_3** Intrinsic value<br>Estimates [*CI*]	**M2.b_3** Attainment value<br>Estimates [*CI*]	**M2.c_3** Utility value<br>Estimates [*CI*]
**Fixed effects**			
Intercept	2.96 [2.851; 3.07]	2.907 [2.804; 3.008]	2.907 [2.804; 3.008]
Learning situation-level			
Detail	.021 [-.039; .081]	.016 [-.031; .063]	.016 [-.031; .063]
Variation	-.049 [-.118; .018]	-.004 [-.056; .050]	-.004 [-.056; .050]
Logical Inconsistency	-.041 [-.185; .102]	-.074 [-.186; .041]	-.074 [-.186; .041]
**Random parameters**			
σ^2 (response-level)	.304 [.285; .325]	.273 [.256; .291]	.273 [.256; .291]
σ^2 (student-level)	.114 [.084; .154]	.183 [.141; .242]	.183 [.141; .242]
σ^2 (learning situation-level)	.022 [.012; .037]	.010 [.004; .019]	.010 [.004; .019]
ICC			
student-level	.258 [.202; .324]	.394 [.33; .463]	.394 [.330; .463]
learning situation-level	.049 [.028; .081]	.021 [.008; .041]	.021 [.008; .041]
ppp-value	.473 [-11.970; 12.969]	.469 [-11.778; 12.923]	.479 [-12.519; 13.244]
	**M3.a_3** Effort costs<br>Estimates [*CI*]	**M3.b_3** Emotional costs<br>Estimates [*CI*]	**M3.c_3** Opportunity costs<br>Estimates [*CI*]
**Fixed effects**			
Intercept	1.933 [1.798; 2.071]	1.613 [1.505; 1.722]	1.76 [1.632; 1.889]
Learning situation-level			
Detail	-.017 [-.083; .048]	-.011 [-.063; .041]	-.012 [-.059; .035]
Variation	.053 [-.021; .127]	.026 [-.030; .082]	.033 [-.020; .086]
Logical Inconsistency	.033 [-.122; .189]	-.033 [-.153; .086]	.004 [-.109; .118]
**Random parameters**			
σ^2 (response-level)	.367 [.343; .392]	.283 [.266; .303]	.266 [.249; .285]
σ^2 (student-level)	.306 [.236; .403]	.198 [.151; .260]	.407 [.319; .528]
σ^2 (learning situation-level)	.025 [.014; .042]	.013 [.006; .023]	.010 [.004; .019]
ICC			
student-level	.438 [.373; .509]	.400 [.335; .470]	.595 [.533; .659]
learning situation-level	.035 [.020; .059]	.026 [.012; .047]	.014 [.006; .028]
ppp-value	.485 [-13.177; 13.107]	.469 [-12.744; 12.525]	.471 [-12.507; 12.986]

Note. For the fixed effect estimates, cell entries are parameter (beta) estimates and CI = 95% credibility intervals. Random effects are presented as estimates and credibility intervals. The *ppp*-value refers to posterior predictive p-value, a measure of model fit.

Table C.4.4 *Zero-order correlations among students' situational motivation and instructional clarity at learning situation-level 2b*

Model		Detail	Variation	Logical Inconsistency
		Estimates [*CI*]	Estimates [*CI*]	Estimates [*CI*]
M1.a_3	Expectation of success	.348 [.012; .641]	.072 [-.261; .398]	.123 [-.235; .451]
M1.b_3	Competence beliefs	.019 [-.267; .298]	-.176 [-.440; .120]	-.139 [-.412; .161]
M2.a_3	Intrinsic value	.109 [-.170; .374]	-.210 [-.463; .077]	-.082 [-.361; .212]
M2.b_3	Attainment value	.123 [-.213; .451]	-.012 [-.334; .316]	-.222 [-.551; .133]
M2.c_3	Utility value	.084 [-.221; .371]	-.079 [-.362; .229]	-.157 [-.452; .160]
M3.a_3	Effort costs	-.072 [-.345; .216]	.214 [-.073; .469]	.066 [-.228; .353]
M3.b_3	Emotional costs	-.068 [-.377; .240]	.148 [-.172; .449]	-.087 [-.397; .244]
M3.c_3	Opportunity costs	-.085 [-.400; .248]	.221 [-.110; .520]	.019 [-.326; .358]

Note. CI = Credibility Interval.

References

Asparouhov, T., & Muthén, B. (2010). *Bayesian analysis of latent variable models using Mplus*. Muthén & Muthén. http://www.statmodel2.com/download/BayesAdvantages18.pdf

Gelman, A., & Rubin, D. B. (1992). Inference from iterative simulation using multiple sequences. *Statistical Science*, 7, 457-511. https://doi.org/10.1214/ss/1177011136

Gelman, A., Carlin, J. B., Stern, H. S., & Rubin, D. B. (2004). *Bayesian data analysis* (2nd ed.). Chapman & Hall. https://doi.org/10.1201/9780429258411

Hox, J. J., Moerbeek, M., & Schoot, R. v. d. (2018). *Multilevel analysis: techniques and applications* (3rd ed.). Routledge.

Muthén, B., & Asparouhov, T. (2012). Bayesian structural equation modeling: a more flexible representation of substantive theory. *Psychological Methods*, *17*(3), 313-335. https://doi.org/10.1037/a0026802

Muthén, L. K., & Muthén, B. O. (1998-2017). *Mplus User's Guide. Eighth Edition.* Muthén & Muthén.

Van de Schoot, R., Kaplan, D., Denissen, J., Asendorpf, J. B., Neyer, F. J., & Van Aken, M. A. (2014). A gentle introduction to Bayesian analysis: Applications to developmental research. *Child development*, *85*(3), 842-860. https://doi.org/10.1111/cdev.12169
